# The Deubiquitinase OTULIN Is an Essential Negative Regulator of Inflammation and Autoimmunity

**DOI:** 10.1016/j.cell.2016.07.019

**Published:** 2016-08-25

**Authors:** Rune Busk Damgaard, Jennifer A. Walker, Paola Marco-Casanova, Neil V. Morgan, Hannah L. Titheradge, Paul R. Elliott, Duncan McHale, Eamonn R. Maher, Andrew N.J. McKenzie, David Komander

**Affiliations:** 1 Medical Research Council Laboratory of Molecular Biology, Francis Crick Avenue, Cambridge Biomedical Campus, Cambridge CB2 0QH, UK; 2 Institute of Cardiovascular Sciences, College of Medical and Dental Sciences, University of Birmingham, Birmingham B15 2TT, UK; 3 Department of Clinical Genetics, Birmingham Women’s NHS Foundation Trust, Birmingham B15 2TG, UK; 4 Institute of Cancer and Genomic Sciences, University of Birmingham, Birmingham B15 2TT, UK; 5 New Medicines, UCB Pharma, Slough SL1 3WE, UK; 6 Department of Medical Genetics, University of Cambridge and Cambridge NIHR Biomedical Research Centre, Cambridge Biomedical Campus, Cambridge CB2 0QQ, UK

## Abstract

Methionine-1 (M1)-linked ubiquitin chains regulate the activity of NF-κB, immune homeostasis, and responses to infection. The importance of negative regulators of M1-linked chains in vivo remains poorly understood. Here, we show that the M1-specific deubiquitinase OTULIN is essential for preventing TNF-associated systemic inflammation in humans and mice. A homozygous hypomorphic mutation in human *OTULIN* causes a potentially fatal autoinflammatory condition termed OTULIN-related autoinflammatory syndrome (ORAS). Four independent OTULIN mouse models reveal that OTULIN deficiency in immune cells results in cell-type-specific effects, ranging from over-production of inflammatory cytokines and autoimmunity due to accumulation of M1-linked polyubiquitin and spontaneous NF-κB activation in myeloid cells to downregulation of M1-polyubiquitin signaling by degradation of LUBAC in B and T cells. Remarkably, treatment with anti-TNF neutralizing antibodies ameliorates inflammation in ORAS patients and rescues mouse phenotypes. Hence, OTULIN is critical for restraining life-threatening spontaneous inflammation and maintaining immune homeostasis.

## Highlights

• Mutation of OTULIN causes OTULIN-related autoinflammatory syndrome (ORAS) in humans
• Anti-TNF treatment reverses inflammation in ORAS patient and OTULIN-deficient mice
• OTULIN deficiency deregulates M1-polyUb signaling and causes sterile inflammation
• Loss of OTULIN has cell-type-specific effects on LUBAC abundance and signaling

## Graphical Abstract

**Figure:**
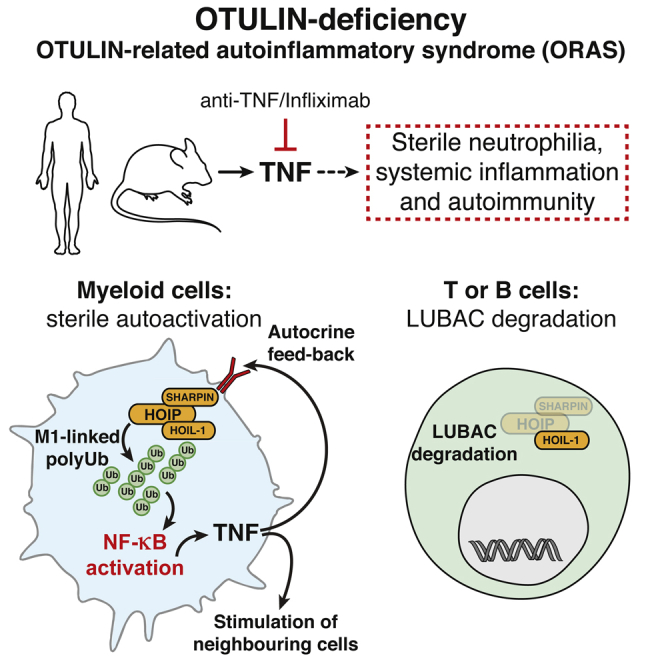

## Teaser

A homozygous hypomorphic mutation in the deubiquitinase OTULIN causes a potentially fatal autoinflammatory disorder, which is reconciled in mouse models.

## Introduction

Protein ubiquitination regulates virtually every aspect of cellular homeostasis, in large part through structurally and functionally distinct polyubiquitin (polyUb) signals (Komander and Rape, 2012). PolyUb chains can be linked via one of seven Ub Lys (K) residues (e.g., K63-linked chains) or via Ub Met1 (M1), forming M1-linked (also known as linear) chains. The latter have important roles in regulating the immune system, in which they contribute to regulating nuclear factor-κB (NF-κB) transcription factors that orchestrate immune responses (Bonizzi and Karin, 2004).

Ub chains regulate canonical NF-κB activation by mediating timed degradation of the inhibitor of κB (IκB) proteins but also serve as a scaffolding, recruitment, and activation platform in receptor signaling complexes. Non-degradative K63- and M1-linked chains mediate the key upstream event of recruiting the TGFβ-activated kinase (TAK1) and the IκB kinase (IKK) complexes, respectively (Jiang and Chen, 2012). K63 and M1 linkages occur in the same Ub polymers (Emmerich et al., 2013), facilitating TAK1 and IKK co-localization and cross-activation.

M1-linked chains are generated by the linear ubiquitin chain assembly complex (LUBAC) consisting of HOIP, HOIL-1, and SHARPIN (Fiil and Gyrd-Hansen, 2014, Iwai et al., 2014). LUBAC is recruited to many immune receptors, including TNF-R1, IL-1R, CD40, TLRs, and NOD2, in a Ub-dependent manner. At the receptors, LUBAC ubiquitinates a host of targets, including RIPK1, RIPK2, MyD88, IRAKs, and NEMO, directly or on pre-existing Ub chains (Fiil and Gyrd-Hansen, 2014, Iwai et al., 2014).

Genetic loss of LUBAC components leads to immunodeficiency (MacDuff et al., 2015) and inflammatory phenotypes in mice (Gerlach et al., 2011, Ikeda et al., 2011, Tokunaga et al., 2011, Tokunaga et al., 2009), which can be rescued by co-deletion of TNF-R1 (Gerlach et al., 2011, Kumari et al., 2014, Peltzer et al., 2014, Rickard et al., 2014). Mutations in LUBAC components also cause inflammatory conditions in humans (Boisson et al., 2015, Boisson et al., 2012). Hence, loss of M1-linked chains imbalances immune signaling.

Several deubiquitinating enzymes (DUBs), including A20, CYLD, and Cezanne, act as negative regulators of NF-κB signaling and are essential for resolving inflammation and the return to homeostasis (Harhaj and Dixit, 2012). OTULIN (also known as FAM105B or Gumby) is the only DUB known to specifically cleave M1 linkages (Keusekotten et al., 2013, Rivkin et al., 2013). OTULIN directly binds the LUBAC component HOIP, and knockdown of OTULIN in human cell lines increases M1-linked chains on LUBAC and its substrates (Elliott et al., 2014, Fiil et al., 2013, Keusekotten et al., 2013, Rivkin et al., 2013, Schaeffer et al., 2014). Strikingly, while LUBAC translocates to receptor signaling complexes (RSCs), OTULIN is not stably associated with TNF or NOD2 RSCs (Draber et al., 2015), and how it regulates signaling complexes, e.g., TNF signaling, is unclear (Hrdinka et al., 2016). Indeed, the physiological role of OTULIN in the immune system has remained unstudied, since OTULIN loss-of-function mutations lead to early embryonic lethality (E12.5–E14) in mice due to defective Wnt signaling and angiogenesis (Rivkin et al., 2013).

Here, we describe that a homozygous hypomorphic OTULIN mutation in a consanguineous family causes a potentially fatal autoinflammatory disorder termed OTULIN-related autoinflammatory syndrome (ORAS), which can be managed by Infliximab (anti-TNF neutralizing antibody). We recapitulate key features of ORAS in mouse models of OTULIN deficiency. In an acute model, induced loss of OTULIN in immune cells leads to multi-organ inflammation and deterioration of animals within a few days; this can be ameliorated by anti-TNF, but not by neutralization of other upregulated cytokines. In addition, loss of OTULIN in myeloid cells generates a chronic model in which mice display increased serum levels of inflammation-associated cytokines and chemokines and develop splenomegaly and autoimmunity. In bone-marrow-derived macrophages (BMDMs), loss of OTULIN leads to overproduction of M1-linked Ub chains and dysregulated NF-κB activation and cytokine secretion. Strikingly, while mice lacking OTULIN in B or T cells do not display overt inflammatory phenotypes, further analysis indicates that these OTULIN-deficient cells have downregulated LUBAC components HOIP and SHARPIN, but not HOIL-1.

Together, the data from mouse models and human patients clearly establish OTULIN and M1-linked polyUb chains as key regulators of immune homeostasis, inflammation, and autoimmunity and reveal cell-type-specific effects of OTULIN in immune cells.

## Results

### Hypomorphic OTULIN Germline Mutation in Patients with Idiopathic Inflammatory Disease

**Figure 1:**
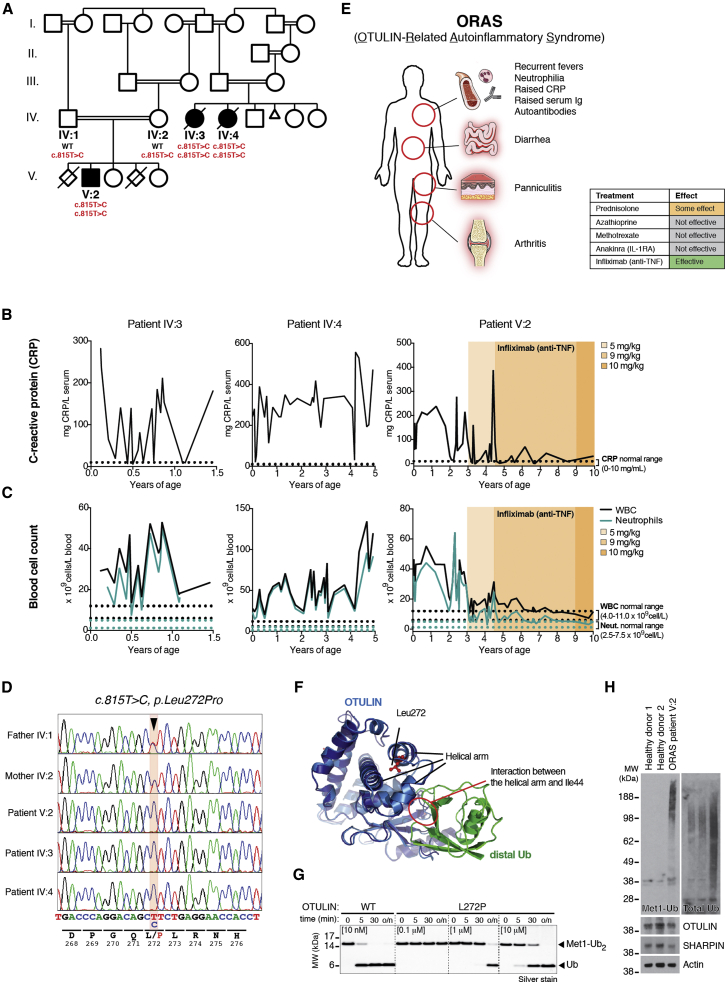
Mutations in *OTULIN* in Patients with a Systemic Autoinflammatory Syndrome (A) Segregation of the inflammatory symptoms (filled symbols) and the *c.815T>C* substitution in *OTULIN* in the affected kindred. ○, females; □, males; double lines, consanguineous relationship; crossed symbols, deceased individuals; Δ, miscarriage; ◊, stillbirths. Roman numerals indicate generations. (B and C) Lifetime measurements of (B) C-reactive protein (CRP) serum concentrations and (C) white blood cell (WBC, black line) and neutrophil numbers (cyan line) in blood from patients IV:3, IV:4, and V:2. Reference ranges (dotted lines) are indicated on the graphs. Patient V:2 was treated with Infliximab as indicated (orange shade). (D) *OTULIN* DNA sequence chromatograms identifying the homozygous single-base substitution (*c.815 T>C, p.Leu272Pro*, arrowhead). (E) Schematic of the cardinal symptoms of OTULIN-related autoinflammatory syndrome (ORAS). The efficacy of trialed treatments are indicated in the table. (F) Superimposed structures of OTULIN’s catalytic domain (blue) without substrate and bound to Met1 diUb (green [only distal Ub shown]; PDB: 3znv and PDB: 3znz [Keusekotten et al., 2013]), showing the position of Leu272 in the distal Ub binding site. (G) Met1-linked diUb hydrolysis by OTULIN^WT^ and OTULIN^L272P^. (H) Immunoblot showing the levels of Met1-linked polyUb, total Ub, OTULIN, and LUBAC in buffy coat cells from patient V:2. See also Figure S1 and Document S1. Tables S1 and S3–S8, Table S2. Rare Homozygous Variants in ORAS Patients, Related to Figure 1.

From the late 1990s, three premature newborns (born at week 34, week 36, and week 28+6, respectively) from a consanguineous family (Figure 1A) displayed severe idiopathic inflammatory symptoms. Within days to weeks after birth, they had repeated episodes of systemic inflammation with diarrhea and elevated serum C-reactive protein (CRP; Figure 1B) and white blood cell (WBC) and neutrophil count (Figure 1C) without evidence of infection. All three eventually developed relapsing nodular panniculitis with neutrophil infiltrate and recurrent fevers. The patients showed reduced growth parameters and also exhibited painful swollen joints as well as elevated immunoglobulin levels and autoantibodies in serum (Table S1 and Experimental Models and Subject Details). Genetic testing excluded Mediterranean fever, chronic infantile neurological cutaneous articular syndrome, tumor necrosis factor-receptor associated periodic syndrome, and hyper IgD syndrome as the cause of the symptoms (Experimental Models and Subject Details).

**Figure S1:**
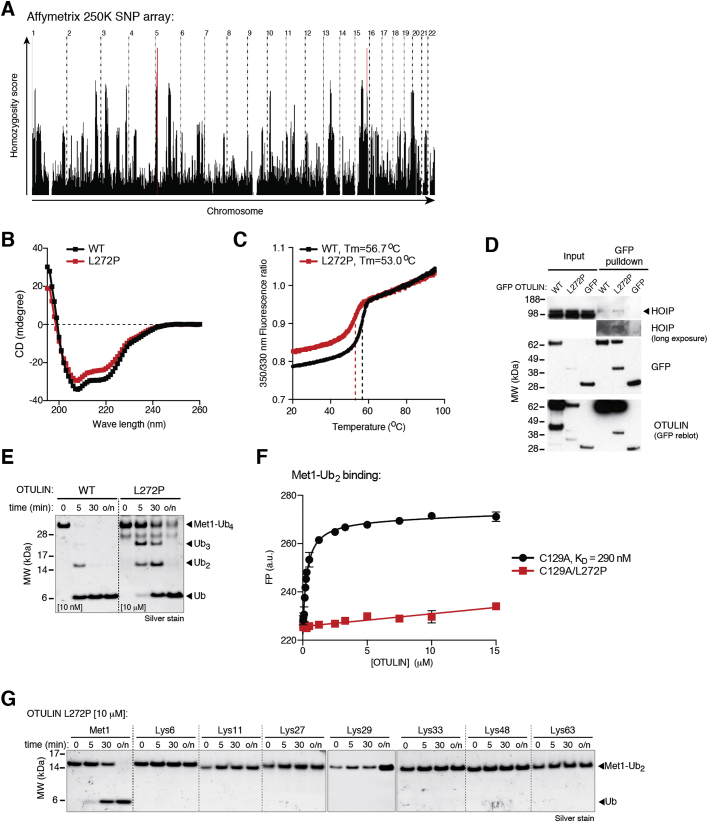
Genetic Linkage Analysis of Affected Patients and Biochemical and Biophysical Analysis of OTULIN^L272P^, Related to Figure 1 (A) Candidate regions of linkage following genome-wide linkage scan using Affymetrix 250K SNP arrays in three affected patients. HomozygosityMapper output shows common regions of genome-wide homozygosity in the three patients. The graph shows the genome-wide homozygosity scores produced by HomozygosityMapper plotted as bar chart with red bars indicating the most promising genomic linkage regions (red). (B) Circular dichroism spectroscopy in the ultraviolet wavelength region of OTULIN^WT^ and OTULIN^L272P^. This spectrum is representative of two independent experiments carried out at a protein concentration of 0.38 mg/mL. (C) Tryptophan fluorescence upon thermal unfolding measured by nanoDSF. Destabilization of OTULIN^WT^ and OTULIN^L272P^ dependent on temperature shows that both proteins are stable at 37°C. Apparent melting temperatures (T_m_) were determined as 56.7°C and 53°C, respectively (dashed lines). Data are representative of two independent experiments. (D) Immunoblot of endogenous HOIP co-precipitating with C-terminally GFP-tagged OTULIN^WT^ or OTULIN^L272P^ ectopically expressed in HEK293 cells. Data are representative of two independent experiments. (E) Met1-linked tetraUb hydrolysis by OTULIN^WT^ and OTULIN^L272P^ were assayed with 1 μM tetraUb over time with the indicated OTULIN concentrations and visualized on silver-stained 4%–12% gradient SDS-PAGE gels. (F) Affinity measurements by fluorescence anisotropy with FlAsH (Fluorescein Arsenical Helix-binder)-labeled Met1 diUb and catalytically inactive OTULIN^C129A^ or OTULIN^C129A/L272P^. Data are means ± SD of one experiment performed in triplicate. Results are representative of three independent experiments. FP, fluorescence polarization. Values were fitted to a one-site total binding model to derive binding constants (K_D_), which could not be calculated for OTULIN^C129A/L272P^. (G) Hydrolysis of diUb of all possible linkages by OTULIN^L272P^ in a time course with the indicated OTULIN^L272P^ concentration and visualized on silver-stained 4%–12% gradient SDS-PAGE gels. o/n, overnight treatment.

SNP array analysis of the three affected individuals identified three regions of extended homozygosity at chromosomes 5 and 15 shared by all three individuals (Figure S1A and Tables S2 and S3). The largest region of shared homozygosity was at chromosome 5p15 from 13,802,063 to 16,722,976 bp between rs795541 and rs4702171. Genotyping of all available family members with microsatellite markers D5S817, D5S1991, D5S1992, D5S1963, and D5S416 confirmed linkage to this candidate region. Within the ∼3 Mb candidate region of chromosome 5p15, sequencing of *FAM105A*, *FBXL7*, *MARCH11*, *ZNF622*, and *FAM34B* revealed no candidate pathogenic variants. However, we detected a homozygous missense substitution in *OTULIN/FAM105B* in all affected individuals (*c.815T>C*; *p.Leu272Pro*) (Figure 1D). The parents of patient V:2 (IV:1 and IV:2) were both heterozygous for the substitution (Figure 1D), and none of the healthy siblings were homozygous for the *c.815T>C* variant (data not shown). Moreover, the variant was not present in the ∼12,000 and ∼120,000 alleles represented in the Exome Variant Server and EXaC datasets, respectively, either. Consistently, whole-exome sequencing of patient V:2 revealed no other homozygous or previously annotated pathogenic variants likely to cause the disease phenotype (Tables S2 and S3). Thus, we termed the described syndrome OTULIN-related autoinflammatory syndrome (ORAS) (Figure 1E).

Leu272 is located in a helix of the catalytic OTU domain that forms part of the binding pocket for M1-linked diUb, and mutation to Pro would be predicted to disrupt OTULIN Ub binding (Figure 1F). Recombinant OTULIN^L272P^ displayed small differences in overall fold and reduced thermal stability (Figures S1B and S1C), and expression of OTULIN^L272P^ in HEK293 cells consistently led to significantly reduced levels of OTULIN^L272P^ as compared to wild-type protein (Figure S1D). OTULIN^L272P^, although less stable, maintained its interaction with HOIP (Figure S1D).

In addition to reduced stability, OTULIN^L272P^ was 1,000–10,000 times less active toward M1-linked di- and tetraUb (Figures 1G and S1E). M1-diUb binding to catalytically inactive OTULIN^C129A/L272P^ was significantly diminished yet not completely abrogated (Figure S1F). OTULIN^L272P^ was still M1-linkage specific (Figure S1G), as the affected Ub binding site does not dictate OTULIN specificity (Keusekotten et al., 2013).

Reduced stability and activity of OTULIN suggested an impact on M1-linked polyUb in ORAS patients. Patient blood samples confirmed the presence of OTULIN, albeit at slightly reduced levels as compared to control samples, as well as the presence of SHARPIN (Figure 1H). Strikingly, while samples from age-matched controls showed barely detectable levels of M1-linked chains, this chain type was strongly increased in the ORAS patient sample.

We conclude that loss of OTULIN function and increased levels of M1-linked polyUb in human cells may indeed lead to the severe inflammation and autoimmunity observed in ORAS.

### Neutralization of TNF Ameliorates Systemic Inflammation in ORAS

Importantly, ORAS can be managed by treatment with the anti-TNF antibody Infliximab. Treatment of affected patients with prednisolone (general corticosteroidal immunosuppressant), azathioprine, and methotrexate (anti-proliferative immunosuppressive drugs), as well as Anakinra (recombinant IL-1R-antagonist), had little or no effect on the symptoms (Figure 1E and Experimental Models and Subject Details). Patient IV:3 died at 16 months of age of pneumococcal septicemia, and patient IV:4 died from an episode of systemic inflammation leading to acute renal failure and pulmonary edema at age 5. Of note, neither patient IV:3 nor IV:4 were treated with Infliximab. By contrast, patient V:2 responded well to treatment with Infliximab (Figures 1B and 1C) and is currently alive (age 11) with well-controlled disease. Infliximab treatment drastically reduced the symptoms, and serum levels of CRP as well as WBC and neutrophil counts in blood, returned to normal ranges after treatment (Figures 1B, 1C), suggesting a TNF-mediated pathogenesis in the patients.

### Deletion of *Otulin* in Mouse Immune Cells Drives Acute Systemic Inflammation

**Figure 2:**
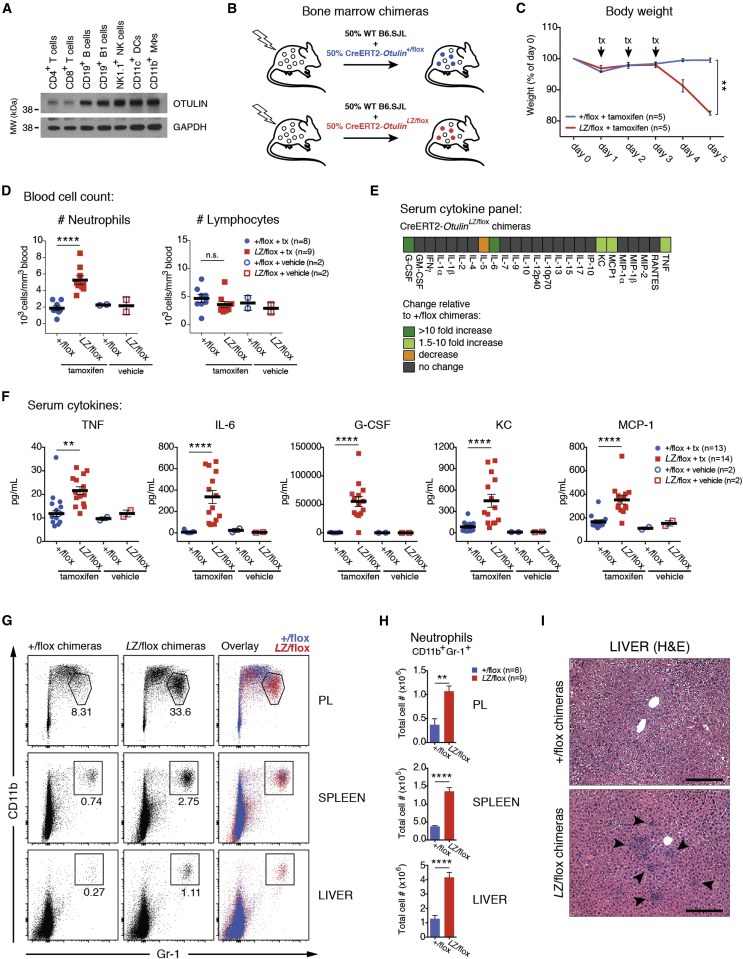
Deletion of *Otulin* in Immune Cells Causes Acute Systemic Inflammation in Mice (A) OTULIN immunoblot on immune cells from wild-type mice. NK cell, natural killer cell; DC, dendritic cell; MΦ, macrophage. (B) Schematic representation of mixed bone marrow chimera generation. Wild-type (WT) B6.SJL cells are CD45.1^+^, and CreERT2-*Otulin*^flox^ cells are CD45.2^+^. (C) Body weight following i.p. administration of tamoxifen (tx; arrows) to CreERT2-*Otulin*^flox^ chimeric mice. (D) Neutrophil and lymphocyte counts from blood of CreERT2-*Otulin*^flox^ chimeras and vehicle-treated controls at day 5 following tamoxifen administration. (E and F) Luminex multiplex analysis of serum cytokines and chemokines from terminal bleeds on day 5 presented as (E) a heatmap of relative changes in concentration of all analytes between CreERT2-*Otulin*^+/flox^ and CreERT2-*Otulin*^*Lac*Z/flox^ chimeras and (F) serum concentrations of cytokines and chemokines increased in CreERT2-*Otulin*^*Lac*Z/flox^ chimeras. Data were pooled from two independent experiments. (G and H) Flow cytometry analysis of CD11b^+^Gr-1^+^ neutrophils in total cellular infiltrate (CD45.1^+^ and CD45.2^+^) in peritoneal lavage (PL), spleen, and liver from CreERT2-*Otulin*^flox^ chimeras presented as (G) representative dot plots with percentage of cells in gate indicated and (H) total cell number. (I) Micrographs of hematoxylin and eosin (H&E) stained sections reveal inflammatory foci (arrowheads) in liver parenchyma. Micrographs are representative of 13 CreERT2-*Otulin*^+/flox^ and 14 CreERT2-*Otulin*^*Lac*Z/flox^ chimeras from two independent experiments. Scale bars, 200 μm. (C, D, F, and H) Data are presented as mean ± SEM, and n represents number of mice. See also Figure S2 and Table S4.

**Figure S2:**
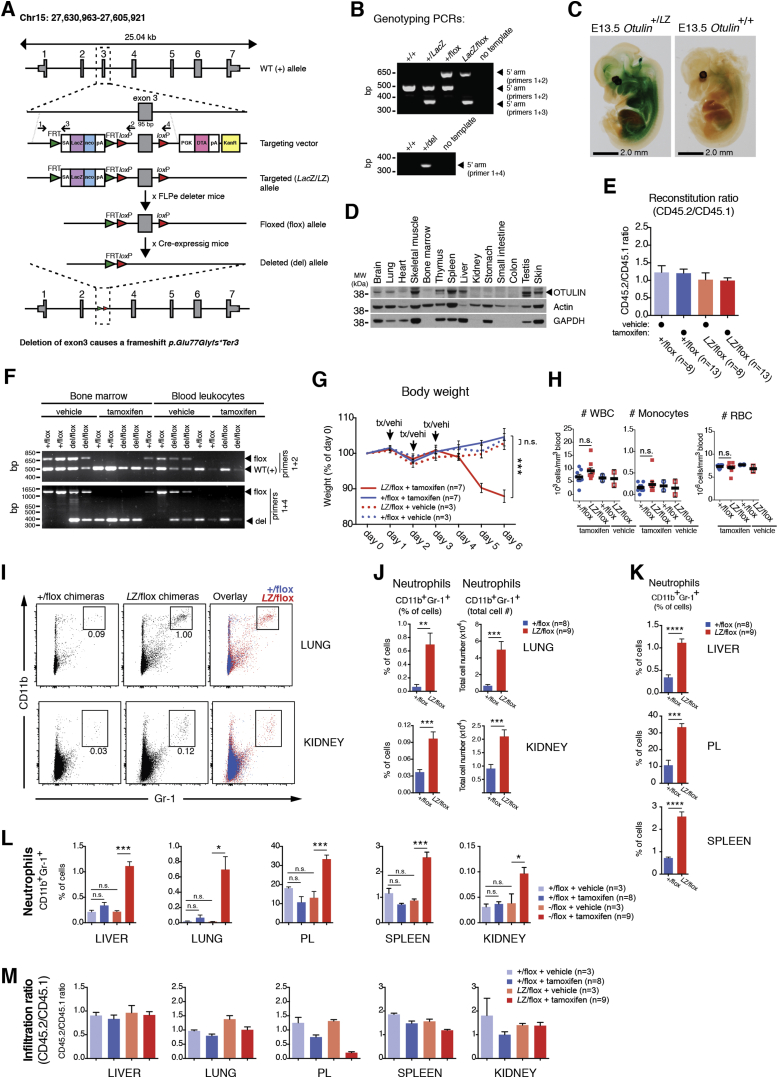
Generation of OTULIN-Targeted Mice, OTULIN Expression, Genotyping, and Reconstitution of Bone Marrow Chimeric Mice, Related to Figure 2 (A) Schematic showing the strategy to generate conditional and cell-type-specific knockouts of *Otulin*. SA, splice acceptor; neo, neomycin-resistance cassette; pA, polyA signal; PGK, murine PGK-1 promoter; DTA, diphtheria toxin A selection cassette; KanR, Kanamycin-resistance cassette. (B) Genotyping of mouse strains. PCR reactions showing the expected products from each genotype. (C) E13.5 embryos stained with X-gal for β-galactosidase activity and cleared by methyl salicylate shows *Otulin* promoter activity in multiple tissues. Pictures are representative of five embryos of each genotype from two independent experiments. (D) Immunoblot analysis showing OTULIN expression in multiple tissues from adult wild-type C57BL/6 mice. Blots are representative of three independent experiments. (E) Ratio of CD45.1^+^ (wild-type B6.SJL) and CD45.2^+^ (CreERT2-*Otulin*^+/flox^ or CreERT2-*Otulin*^*Lac*Z/flox^ C57BL/6) expressing splenocytes determined by flow cytometry at the termination of chimera experiments. Data were pooled from two independent experiments. (F) Genotyping of bone marrow cells or blood leukocytes from CreERT2-*Otulin*^flox^ chimeras treated with tamoxifen or vehicle shows complete or near-complete conversion of flox alleles to del alleles upon tamoxifen treatment. Note that WT(+) products are present in all reactions as BJ6.SJL WT cells are present in all samples from the chimeras. (G) Body weight following i.p. administration of tamoxifen (tx) or vehicle (vehi) to CreERT2-*Otulin*^flox^ chimeric mice. Data were pooled from two independent experiments. (H) Blood cell counts from CreERT2-*Otulin*^flox^ chimeras and vehicle-treated controls at day 5. (I-J) Flow cytometry analysis of CD11b^+^Gr-1^+^ neutrophils in total cellular infiltrate (CD45.1^+^ and CD45.2^+^) in lung and kidney presented as (I) representative dot plots with percentage of cells in gate indicated and (J) total cell number or percentage of cells in gate quantified. (K) Percentage of neutrophils in infiltrate from CreERT2-*Otulin*^flox^ chimeras (related to Figure 2H). Data shown in (J) and (K) are repeated in (L). (L) Flow cytometry analysis of CD11b^+^Gr-1^+^ neutrophils in liver, lung, peritoneal lavage (PL), spleen, and kidney in tamoxifen- or vehicle-treated CreERT2-*Otulin*^flox^ chimeras. (M) Ratio of CD45.1^+^ (wild-type B6.SJL) and CD45.2^+^ (CreERT2-*Otulin*^+/flox^ or CreERT2-*Otulin*^*Lac*Z/flox^ C57BL/6) expressing CD11b^+^Gr-1^+^ neutrophils in each tissue determined by flow cytometry at the termination of chimera experiments. Data are presented as mean ± SEM, and n represents number of mice.

To understand how OTULIN deficiency caused autoinflammation and autoimmunity, we attempted to recapitulate ORAS in mouse models. Due to embryonic lethality (Rivkin et al., 2013), heterozygous *Otulin*^+/*Lac*Z^ mice (Figures S2A and S2B) were bred with appropriate inducible and/or tissue-specific Cre expressing mouse strains. OTULIN was expressed in several tissues in *Otulin*^+/*Lac*Z^ embryos and adult wild-type mice, including spleen and thymus (Figures S2C and S2D). In immune cells, OTULIN was expressed in T cells, B cells, and natural killer (NK) cells and prominently in dendritic cells and macrophages (Figure 2A).

We generated CreERT2-*Otulin*^*Lac*Z/flox^ mice, in which *Otulin* can be ablated in all cells with tamoxifen administration. Tamoxifen administration led to mice becoming moribund within a day (data not shown), suggesting strong phenotypes also in adult mice in addition to reported developmental defects (Rivkin et al., 2013). This prevented further study, and to investigate the role of OTULIN in the immune system, we generated CreERT2-*Otulin*^flox^ mixed bone marrow chimeras (Figure 2B), which were healthy (data not shown) and showed similar levels of CD45.1^+^ and CD45.2^+^ cells in vivo (Figure S2E).

Six to eight weeks after reconstitution, chimeric mice were treated with tamoxifen to induce *Otulin* ablation (Figure S2F). Strikingly, this resulted in rapid weight loss in CreERT2-*Otulin*^*Lac*Z/flox^ chimeras as compared to controls (Figures 2C and S2G). Weight loss was accompanied by a pronounced increase in the number of circulating neutrophils in CreERT2-*Otulin*^*Lac*Z/flox^ chimeras (Figure 2D), while the number of lymphocytes and monocytes and the overall number of blood cells or splenocytes did not change (Figures 2D, S2E, and S2H). We also observed a marked increase in the pro-inflammatory cytokines TNF and IL-6, the neutrophil cytokines G-CSF and KC, and the monocyte/macrophage chemokine MCP-1 in serum of CreERT2-*Otulin*^*Lac*Z/flox^ chimeras compared with CreERT2-*Otulin*^+/flox^ chimeras and vehicle-treated controls (Figures 2E and 2F and Table S4). These cellular and molecular mediators are indicative of an inappropriate and damaging inflammatory response mediated by OTULIN-deficient myeloid cells. As expected in the short timeframe, cytokines associated with adaptive immunity were not elevated (Figure 2E and Table S4).

Necropsy of the chimeras did not reveal any gross anatomical changes, but flow cytometry analysis showed substantial infiltration of CD11b^+^Gr-1^+^ neutrophils in the peritoneal lavage (PL), spleen, and liver and, to some extent, lung and kidney in CreERT2-*Otulin*^*Lac*Z/flox^ chimeras compared with CreERT2-*Otulin*^+/flox^ or vehicle-treated controls (Figures 2G, 2H, and S2I–S2L). Histological analysis confirmed immune cell infiltration in the livers of CreERT2-*Otulin*^*Lac*Z/flox^ chimeras with inflammatory foci scattered in the parenchyma (Figure 2I, arrowheads). With the exception of PL, where neutrophils were recovered in lower numbers after tamoxifen treatment, tissue-infiltrating neutrophils consisted of overall similar numbers of CD45.1^+^ wild-type cells and CD45.2^+^ OTULIN-deficient cells (Figure S2M). This suggests that loss of OTULIN does not cause aberrant cell death in immune cells (Figure S2E) and that the high levels of cytokines and chemokines present activate the entire immune cell population.

**Figure S3:**
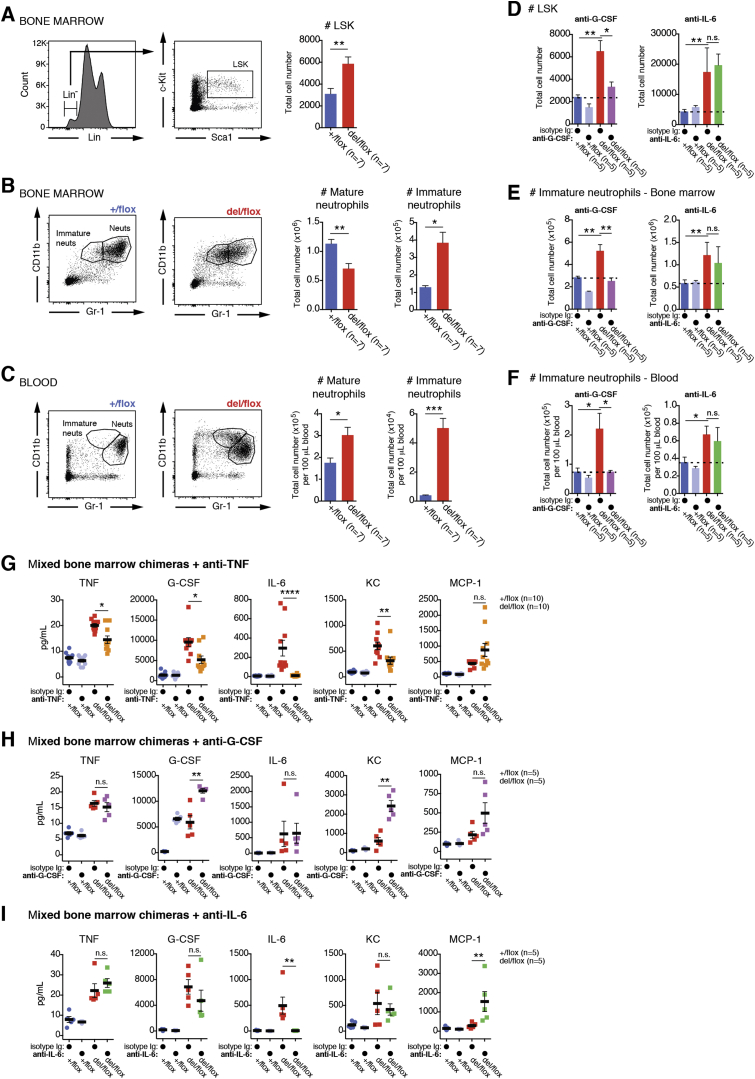
Emergency Granulopoiesis and Neutrophilia Is Controlled by G-CSF in OTULIN-Deficient Mice, Related to Figure 3 (A) Flow cytometry analysis of lineage negative (Lin^-^) c-Kit^+^Sca1^+^ LSK cells in bone marrow of tamoxifen-treated CreERT2-*Otulin*^flox^ chimeric mice shows increased numbers of LSK cells in bone marrow consistent with emergency granulopoiesis. (B-C) Flow cytometry analysis of mature and immature neutrophils in (B) bone marrow and (C) blood of tamoxifen-treated CreERT2-*Otulin*^flox^ chimeric mice shows increased numbers of these cells in both tissues, consistent with emergency granulopoiesis. (D-F) Quantification of flow cytometry analysis as in (A-C) of LSK cells (D) and mature and immature neutrophils in (E) bone marrow and (F) blood of tamoxifen-treated CreERT2-*Otulin*^flox^ chimeric mice injected with anti-G-CSF neutralizing antibodies, anti-IL-6 neutralizing antibodies, or isotype control as indicated. (G-I) Serum concentrations of TNF, G-CSF, IL-6, KC, and MCP-1 from tamoxifen-treated CreERT2-*Otulin*^flox^ chimeric mice injected with (G) anti-TNF neutralizing antibodies, (H) anti-G-CSF neutralizing antibodies, (I) anti-IL-6 neutralizing antibodies, or isotype control as indicated measured by Luminex multiplex analysis. These data are represented as a heat map in Figure 3J. Data are presented as mean ± SEM, and n represents number of mice.

Collectively, this shows that deletion of *Otulin* in immune cells leads to spontaneous and severe acute systemic inflammation characterized by rapid weight loss, increased levels of pro-inflammatory cytokines (in particular, TNF, G-CSF, and IL-6) in serum, neutrophilia with all the hallmarks of emergency granulopoiesis (see Figures S3A–S3C), and infiltration of neutrophils into multiple tissues. This highly pro-inflammatory phenotype of the OTULIN-deficient adult mice is unlikely to arise from defects in Wnt signaling during development (Rivkin et al., 2013) but indeed shows several of the cardinal symptoms of ORAS (Figure 1E).

### Rescue of Systemic Inflammation in OTULIN-Deficient Mice

We next tested whether the strong ORAS-like phenotype can be reversed by antibody treatments, as observed in the human disease. It was unclear whether any of the upregulated cytokines were independently regulated by OTULIN/M1-linked chains. Hence, we tested whether neutralization of TNF, G-CSF, or IL-6 impacted on the weight loss and emergency granulopoiesis observed in CreERT2-*Otulin*^*Lac*Z/flox^ chimeric mice.

**Figure 3:**
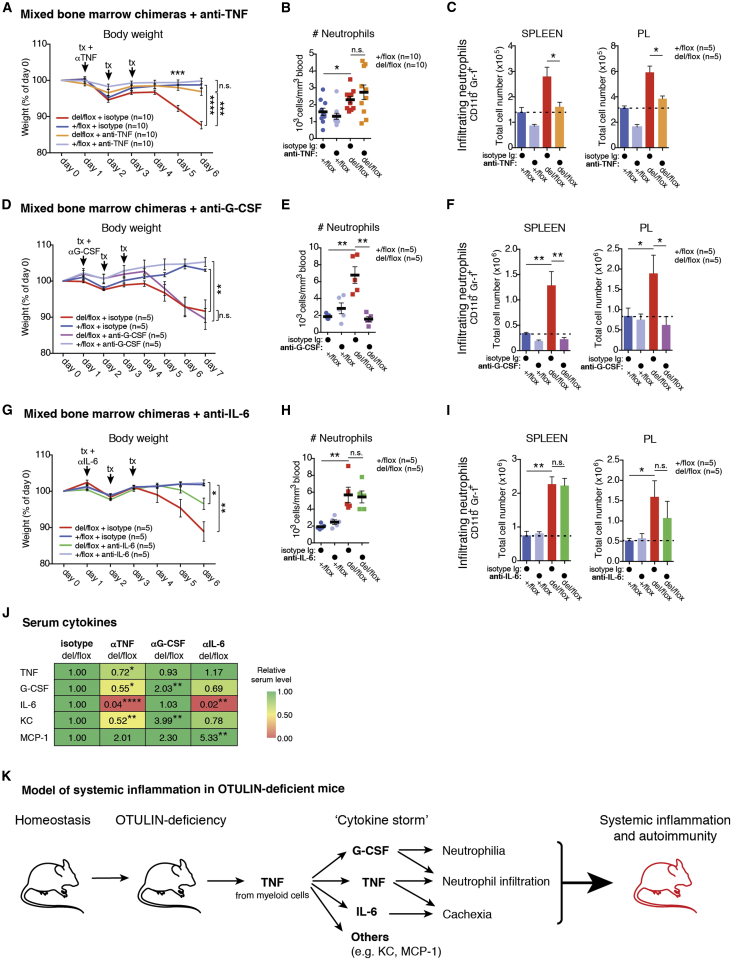
Neutralization of TNF Ameliorates Inflammation Caused by OTULIN Deficiency (A–J) Measurements from tamoxifen (tx)-treated (arrows) CreERT2-*Otulin*^flox^ bone marrow chimeras injected with (A–C) anti-TNF neutralizing antibodies (αTNF) (data were pooled from two independent experiments), (D–F) anti-G-CSF-neutralizing antibodies (αG-CSF), (G–I) anti-IL-6-neutralizing antibodies (αIL-6), or isotype control as indicated. (A, D, and G) Body weight of CreERT2-*Otulin*^flox^ chimeric mice treated with neutralizing antibodies as indicated. (B, E, and H) Blood neutrophil counts from CreERT2-*Otulin*^flox^ chimeric mice treated as indicated. (C, F, and I) Total number of infiltrating CD11b^+^Gr-1^+^ neutrophils in spleen and peritoneal lavage (PL) measured by flow cytometry from CreERT2-*Otulin*^flox^ chimeric mice treated as indicated. (J) Heatmap of Luminex multiplex analysis of cytokines and chemokines in serum from terminal bleeds on days 6 or 7 from chimeric mice treated as indicated. Numbers indicate relative change compared to isotype-treated del/flox mice within each experiment. G-CSF levels for αG-CSF and αIL-6 were measured by ELISA. Asterisks (^∗^) indicate the level of statistical significance. (K) Model of TNF-driven systemic inflammation and the contributions from different cytokines in OTULIN-deficient mice. (A–J) Data are presented as mean ± SEM, and n represents number of mice. See also Figure S3.

In accordance with the effective treatment of ORAS patients with Infliximab, we found that anti-TNF neutralizing antibodies, administered in parallel with tamoxifen-induced *Otulin* deletion, completely ameliorated weight loss (Figure 3A). While the numbers of blood neutrophils were still elevated (Figure 3B), infiltrating neutrophils in spleen and PL of CreERT2-*Otulin*^*Lac*Z/flox^ were reduced to a level comparable to isotype-treated CreERT2-*Otulin*^*+*/flox^ controls (Figure 3C). In contrast, anti-G-CSF or anti-IL-6 were unable to rescue the phenotype fully. While anti-G-CSF treatment strongly reduced blood neutrophil count and infiltration, it failed to rescue weight loss and mice remained cachexic (Figures 3D–3F). Anti-IL-6 treatment had no impact on neutrophilia, but weight loss was partially rescued (Figure 3G–3I). This differential amelioration extended to neutrophil production in the bone marrow, where anti-G-CSF, but not anti-IL-6, reduced the number of uncommitted blood cells (“LSK” cells, for Lin^−^Sca1^+^c-Kit^+^ cells) and immature neutrophils to levels observed in isotype-treated controls (Figures S3D–S3F). This confirms that G-CSF drives emergency granulopoiesis in CreERT2-*Otulin*^*Lac*Z/flox^ chimeras and may contribute to the neutrophilia observed in ORAS patients (Figure 1D). Strikingly, anti-TNF treatment of CreERT2-*Otulin*^*Lac*Z/flox^ chimeras resulted in a global reduction of cytokines, including G-CSF, KC, and TNF itself. IL-6 was reduced to background levels (Figures 3J and S3G). Anti-G-CSF did not affect TNF or IL-6 levels and, in fact, caused elevated production of KC and G-CSF itself (although its functional blockade ameliorated neutrophilia) (Figures 3J and S3H). Anti-IL-6 increased MCP-1 levels, possibly indicating a pathway for compensation in the absence of IL-6 (Figures 3J and S3I).

Together, these data implicate TNF as the pre-eminent factor in driving inflammation in OTULIN-deficient mice, although other cytokines and chemokines clearly contribute to the composite phenotype (Figure 3K). This finding correlates with the fact that anti-TNF treatment reverses the inflammatory symptoms in the ORAS patient and therefore suggests that OTULIN-deficient mice provide a good model to understand the mechanisms underlying ORAS.

### Differential Effects of OTULIN Deletion in Cells of the Immune System

To dissect immune mechanisms contributing to the strong OTULIN deficiency phenotype, we employed mouse models in which *Otulin* can be constitutively ablated in specific immune cell lineages, namely T cells (“CD4Cre” mice, expressing Cre recombinase under control of the *Cd4* promoter), B cells (“MB1Cre” mice, expressing Cre under control of the *Mb1* promoter), and myeloid cells (“LysMCre” mice, expressing Cre under control of the *LysM* promoter, leading to knockout in macrophages, neutrophils, and some dendritic cells).

**Figure 4:**
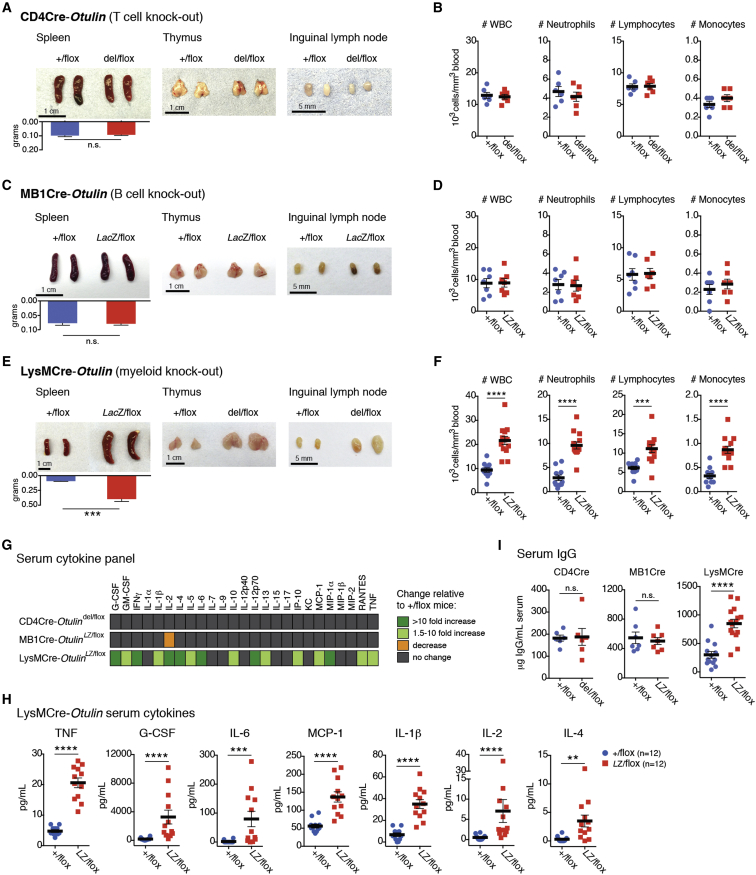
Specific Deletion of *Otulin* in Myeloid Cells, but Not T or B Cells, Causes Systemic Inflammation (A, C, and E) Spleens and spleen weights, thymuses, and inguinal lymph nodes from (A) 2- to 3-months-old CD4Cre-*Otulin*^flox^ mice (n = 6), (C) 3- to 4-months-old MB1Cre-*Otulin*^flox^ mice (n = 4), (E) 3- to 9-months-old LysMCre-*Otulin*^flox^ mice (n = 8). (B, D, and F) Blood cell counts from (B) 2- to 3-months-old CD4Cre-*Otulin*^flox^ mice (n = 6); (D) 3- to 4-months-old MB1Cre-*Otulin*^flox^ mice (n = 7); and (F) 3- to 9-months-old LysMCre-*Otulin*^flox^ mice (n = 11). (G and H) Luminex multiplex analysis of serum cytokine and chemokine concentrations from terminal bleeds of 2- to 3-months-old CD4Cre-*Otulin*^flox^ mice, 3- to 4-months-old MB1Cre-*Otulin*^flox^ mice, and 4- to 9-months-old LysMCre-*Otulin*^flox^ mice presented as (G) a heatmap of relative changes of analytes between OTULIN-deficient mice and their respective +/flox controls and (H) serum concentrations of selected cytokines and chemokines increased in LysMCre-*Otulin*^*Lac*Z/flox^ mice. (I) ELISA measurements of total IgG concentrations in serum from CD4Cre-*Otulin*^flox^ (n = 6), MB1Cre*-Otulin*^flox^ (n = 7), and LysMCre-*Otulin*^flox^ (n = 14) mice. Data are presented as mean ± SEM, and n represents number of mice. See also Figure S4 and Tables S5–S7.

**Figure S4:**
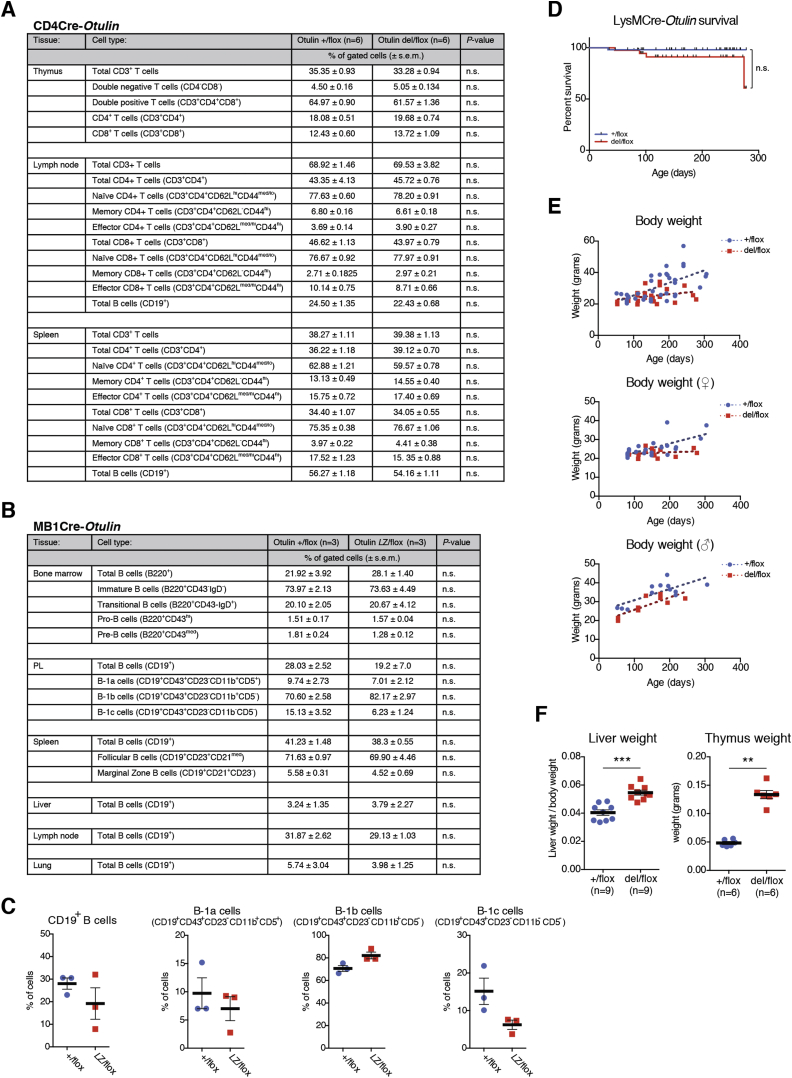
Characterization of CD4Cre-*Otulin*^flox^, MB1Cre-*Otulin*^flox^, and LysMCre-*Otulin*^flox^ mice, Related to Figure 4 (A) Tabulated results from flow cytometry analysis of cellular subsets from thymus, inguinal lymph node, and spleen from CD4Cre-*Otulin*^flox^ mice. (B) Tabulated results from flow cytometry analysis of cellular subsets from bone marrow, peritoneal lavage (PL), spleen, liver, inguinal lymph node, and lung from MB1Cre-*Otulin*^flox^ mice. (C) Results of flow cytometry analysis of peritoneal B cell subsets from MB1Cre-*Otulin*^flox^ mice (n = 3). (D) Kaplan-Meier plot of survival in LysMCre-*Otulin* mice. Censored deaths are indicated as black ticks. (E) Body weight plotted against age in LysMCre-*Otulin*^flox^ mice. Data are presented for either both genders (top panel) or stratified for gender (female (♀), middle panel; male (♂), bottom panel). Dashed lines show the linear regression of the data. (F) Liver weight to body weight ratio (left panel) and thymus weight (right panel) in LysMCre-*Otulin*^flox^ mice. (C and F) Data are presented as mean ± SEM, and n represents number of mice.

Surprisingly, loss of OTULIN in T cells or B cells generated healthy mice with no overt inflammatory phenotypes, as assessed by phenotypic, blood cell, cytokine, and serum IgG analysis (Figures 4 and S4A–S4C and Tables S5 and S6). Closer inspection of the B cell subsets in the MB1Cre-*Otulin*^*Lac*Z/flox^ mice indicated a trend toward lower B1 cell numbers but did not reach statistical significance (Figure S4C).

In stark contrast, deletion of OTULIN in myeloid cells resulted in a strong inflammatory phenotype. LysMCre-*Otulin*^*Lac*Z/flox^ mice were born at Mendelian ratios, appeared healthy at weaning, and showed similar survival as compared to controls but were slightly smaller in terms of body weight (Figures S4D and S4E). Indeed, closer inspection of 3- to 9-month-old LysMCre-*Otulin*^*Lac*Z/flox^ mice revealed a marked increase in the size of lymphoid organs and liver (Figures 4E and S4F) and prominent leukocytosis with increased numbers of circulating neutrophils, lymphocytes, and monocytes (Figures 4F) as compared to controls.

### Deletion of *Otulin* in Myeloid Cells Leads to Chronic Inflammation and Autoimmunity

Strikingly, serum analysis showed that 16 out of 25 tested cytokines and chemokines were markedly elevated in LysMCre-*Otulin*^*Lac*Z/flox^ mice (Figures 4G and 4H and Table S7), including the pro-inflammatory cytokines TNF, IL-6, and IL-1β; the neutrophil and monocyte attractants/activators MCP-1 and MIP-1α; and G-CSF. Interestingly, cytokines associated with T cell activation and adaptive immunity, such as IL-2, IL-4, IFNγ, and RANTES, were also elevated (Figures 4G and 4H and Table S7), as were serum IgG levels (Figure 4I). Collectively, these findings indicate ongoing, systemic inflammation in LysMCre-*Otulin*^*Lac*Z/flox^ mice involving both innate and adaptive immune cells.

**Figure 5:**
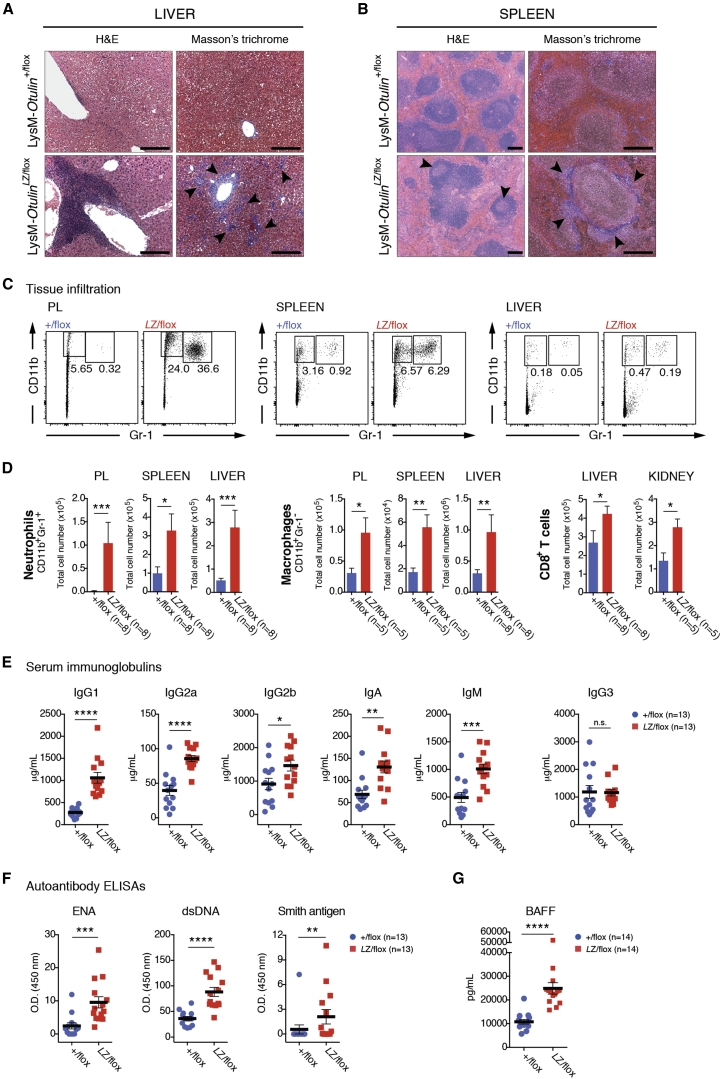
OTULIN Deficiency in Myeloid Cells Leads to Neutrophil Infiltration, Multi-organ Inflammation, Hyper-Immunoglobulinemia, and Autoimmunity (A and B) Micrographs of H&E and Masson’s trichome stained sections of livers and spleens from 4- to 9-months-old LysMCre-*Otulin*^flox^ mice reveal (A) infiltration in and fibrosis (right, arrowheads) of liver parenchyma and (B) distorted spleen architecture with germinal center activation (left, arrowheads) and fibrosis (right, arrowheads) in LysMCre-*Otulin*^*Lac*Z/flox^ mice. Scale bars, 200 μm. Micrographs are representative of five mice of each genotype from two independent experiments. (C and D) Flow cytometry analysis of total cellular infiltrate (CD45.1^+^ and CD45.2^+^) in peritoneal lavage (PL), spleen, liver, and kidney of LysMCre-*Otulin*^flox^ mice presented as (C) representative dot plots of neutrophils with percent of cells in gate indicated and (D) total cell number of neutrophils, macrophages, and CD8^+^ T cells. (E) Concentrations of immunoglobulins in serum from 4- to 9-months-old LysMCre-*Otulin*^flox^ mice. (F) ELISA analysis of serum autoantibody reactivity to ENA, dsDNA, and Smith antigen from 3- to 9-months-old LysMCre-*Otulin*^flox^ mice. (G) ELISA analysis of serum B cell activating factor (BAFF) concentration from 3- to 9-months-old LysMCre-*Otulin*^flox^ mice. (E–G) Data are mean of two technical replicas. (D–F) Data are presented as mean ± SEM, and n represents number of mice. See also Figure S5.

**Figure S5:**
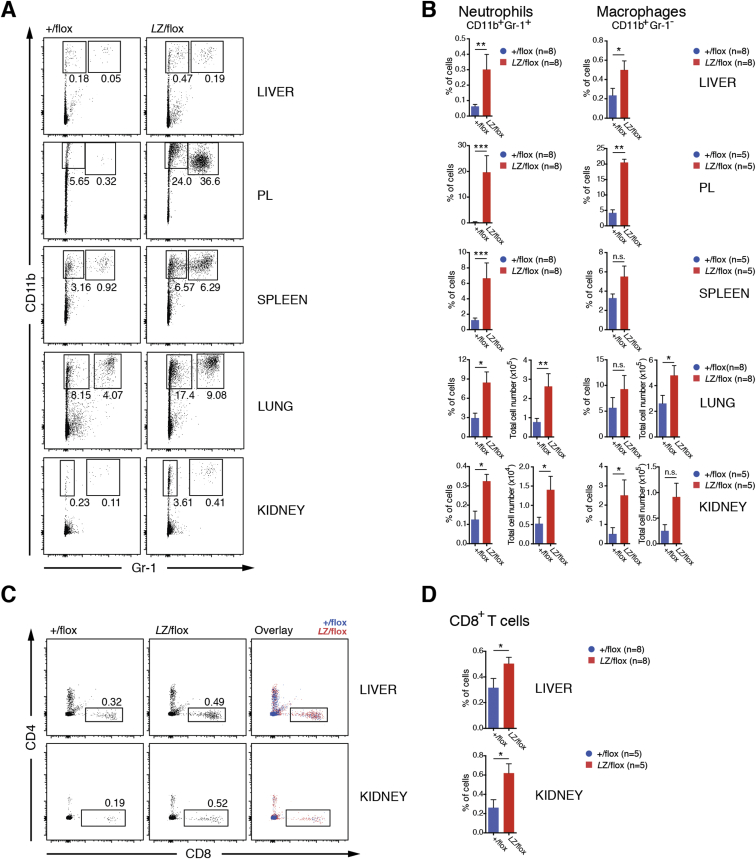
Flow Cytometry Plots and Quantification of Cell Populations in Mice Lacking *Otulin* in Myeloid Cells, Related to Figure 5 (A-D) Flow cytometry analysis of (A-B) CD11b^+^Gr-1^+^ neutrophils and CD11b^+^Gr-1^-^ macrophages, and (C-D) CD8^+^ T cells in liver, peritoneal lavage (PL), spleen, lung, and kidney from from 4-9 month old LysMCre-*Otulin*^flox^ mice presented as (A and C) representative dot plots or (B and D) total cell number or frequency of infiltrating cells. Missing graphs of total number of infiltrating cells in (B) and (D) are shown in main Figure 5D. (A-D) Data were pooled from two independent experiments. Data are presented as mean ± SEM, and n represents number of mice.

Histological analysis revealed substantial immune cell infiltration in livers of LysMCre-*Otulin*^*Lac*Z/flox^ mice, particularly around veins but also in the parenchyma, and a distorted splenic architecture (Figures 5A and 5B). Collagen deposits in liver (Figure 5A, right) and spleen (Figure 5B, right) were visible and consistent with chronic inflammation. Moreover, germinal center activation was evident in LysMCre-*Otulin*^*Lac*Z/flox^ spleens (Figure 5B, left), suggesting B cell hyperactivation and potential for immunoglobulin-mediated pathology. Flow cytometric analysis showed that LysMCre-*Otulin*^*Lac*Z/flox^ mice had greater numbers of both CD11b^+^Gr-1^+^ neutrophils and CD11b^+^Gr-1^−^ macrophages in PL, spleen, liver, lungs, and kidney compared with LysMCre-*Otulin*^*+*/flox^ mice (Figures 5C, 5D, S5A, and S5B). LysMCre-*Otulin*^*Lac*Z/flox^ mice also had increased numbers of CD8^+^ T cells in liver and kidney (Figures 5D, S5C, and S5D), consistent with systemic chronic inflammation and involvement of the adaptive immune system. The number of CD4^+^ T cells remained normal (data not shown).

High-serum IgG and the appearance of active germinal centers in LysMCre-*Otulin*^*Lac*Z/flox^ mice suggested B cell hyperactivation, leading to higher levels of antibodies in the serum, a hallmark of autoimmunity. Indeed, the amounts of serum immunoglobulin isotypes IgG1, IgG2a and b, IgA, and IgM were elevated in LysMCre-*Otulin*^*Lac*Z/flox^ serum, while IgG3 levels were unchanged when compared to LysMCre-*Otulin*^*+/*flox^ (Figure 5E). This indicated polyclonal B cell activation and suggested that LysMCre-*Otulin*^*Lac*Z/flox^ mice could be autoimmune. Consistent with this, ELISA analysis revealed higher antibody reactivity against extractable nuclear antigens (ENA), double-stranded DNA (dsDNA), and Smith antigen in LysMCre-*Otulin*^*Lac*Z/flox^ serum (Figure 5F). B cell activating factor (BAFF) is the primary cytokine that governs peripheral B cell tolerance, and increased BAFF levels can lead to T-cell-independent B cell activation and immunoglobulin production (Groom et al., 2007). Indeed, we found that LysMCre-*Otulin*^*Lac*Z/flox^ mice had elevated levels of BAFF in serum (Figure 5G), suggesting that secretion of BAFF resulting from OTULIN deficiency leads to breakdown of peripheral tolerance and activation of autoreactive B cells.

Collectively, these results show that OTULIN is essential in myeloid cells to prevent unwarranted secretion of cytokines leading to inflammation, as well as autoimmunity (Figure 3K).

### OTULIN Deficiency Leads to Sterile Autoactivation of Inflammatory Pathways

**Figure 6:**
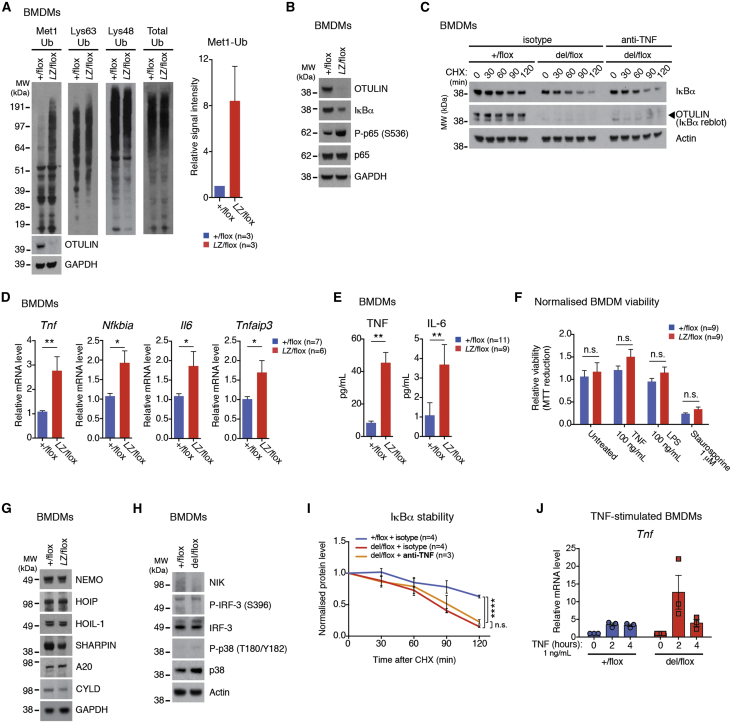
OTULIN Deficiency Leads to Autoactivation of Macrophages (A) Immunoblots of different polyUb chains in whole-cell lysate from untreated (i.e., no exogenous stimulation after differentiation) LysMCre-*Otulin*^flox^ BMDMs. (Right) Densitometry analysis of the Met1-Ub signal from above the 51 kDa marker in immunoblot experiments. (B) Immunoblots of NF-κB signaling proteins from untreated LysMCre-*Otulin*^flox^ BMDMs. (C) Immunoblot analysis of IκBα stability in LysMCre-*Otulin*^flox^ BMDMs treated with anti-TNF neutralizing antibodies or isotype control and cycloheximide (CHX) as indicated. (D) Relative mRNA levels of *Tnf*, *Nfkbia*, *Il6*, and *Tnfaip3* from untreated LysMCre-*Otulin*^flox^ BMDMs measured by quantitative RT-PCR. Each data point is mean of two technical replicas. Statistical significance was determined using two-tailed Student’s *t* test. (E) Luminex analysis of TNF and IL-6 from cell culture supernatants of untreated LysMCre-*Otulin*^flox^ BMDMs. Cells were split, washed in PBS, and reseeded in fresh cell culture medium 24 hr prior to analysis. Results were pooled from two independent experiments. (F) Viability of LysMCre-*Otulin*^flox^ BMDMs 10 hr after treatment. Each experiment was performed as biological duplicates. Results were normalized to LysMCre-*Otulin*^+/flox^. (G and H) Immunoblots of signaling proteins from untreated LysMCre-*Otulin*^flox^ BMDMs. (I) Densitometry analysis of IκBα stability from experiments performed as in (C). (J) Relative mRNA levels of *Tnf* measured by quantitative RT-PCR in LysMCre-*Otulin*^flox^ BMDMs treated with 1 ng/mL TNF as indicated (n = 3). Data are presented as mean ± SEM, and n represents number of biological replicas. See also Figure S6.

We next investigated the molecular mechanisms resulting in the strong inflammatory phenotypes, using BMDMs from LysMCre-*Otulin*^*+*/flox^, LysMCre-*Otulin*^*Lac*Z/flox^, or LysMCre-*Otulin*^del/flox^ mice, which were cultured and studied for pathway activation by western blotting in the absence of any exogenous stimulation. Interestingly, in agreement with the patient samples, the levels of M1-linked Ub chains were markedly increased (∼8-fold on average) in OTULIN-deficient BMDMs, in particular in the high-molecular-weight range, while K63-linked, K48-linked, or total Ub levels were unchanged (Figure 6A). Strikingly, this resulted in NF-κB activation evidenced by degradation of IκBα and increased phosphorylation of p65/RelA in the absence of exogenous stimuli (Figure 6B). Cycloheximide chase experiments confirmed the increased turnover of IκBα (Figure 6C), a hallmark of NF-κB pathway activation. This correlated with increased transcription of NF-κB target genes such as *Tnf*, *Nfkbia (IκBα)*, *Il6*, and *Tnfaip3 (A20)* (Figure 6D) and concomitant secretion of TNF and IL-6 (Figure 6E). Also, LysMCre-*Otulin*^*+*/flox^ and LysMCre-*Otulin*^*Lac*Z/flox^ BMDMs were similarly viable when left untreated or when treated with LPS, TNF, or Staurosporine (Figure 6F), suggesting no apparent sensitization to cell death in these cells.

Importantly, while SHARPIN levels were slightly reduced, the levels of HOIP and HOIL-1 were unchanged in LysMCre-*Otulin*^*Lac*Z/flox^ BMDMs (Figure 6G), consistent with strong production of M1-linked polyUb in these cells (Figure 6A). Protein levels of NEMO/IKKγ, A20, and CYLD were similar to controls (Figure 6G). LUBAC signaling may impact alternative inflammatory pathways, yet in LysMCre-*Otulin*^del/flox^ BMDMs, NF-κB-inducing kinase (NIK) was not stabilized (indicating no activation of non-canonical NF-κB), and IRF-3 or p38 phosphorylation was not increased (suggesting that IRF or MAPK signaling pathways were not active) (Figure 6H).

**Figure S6:**
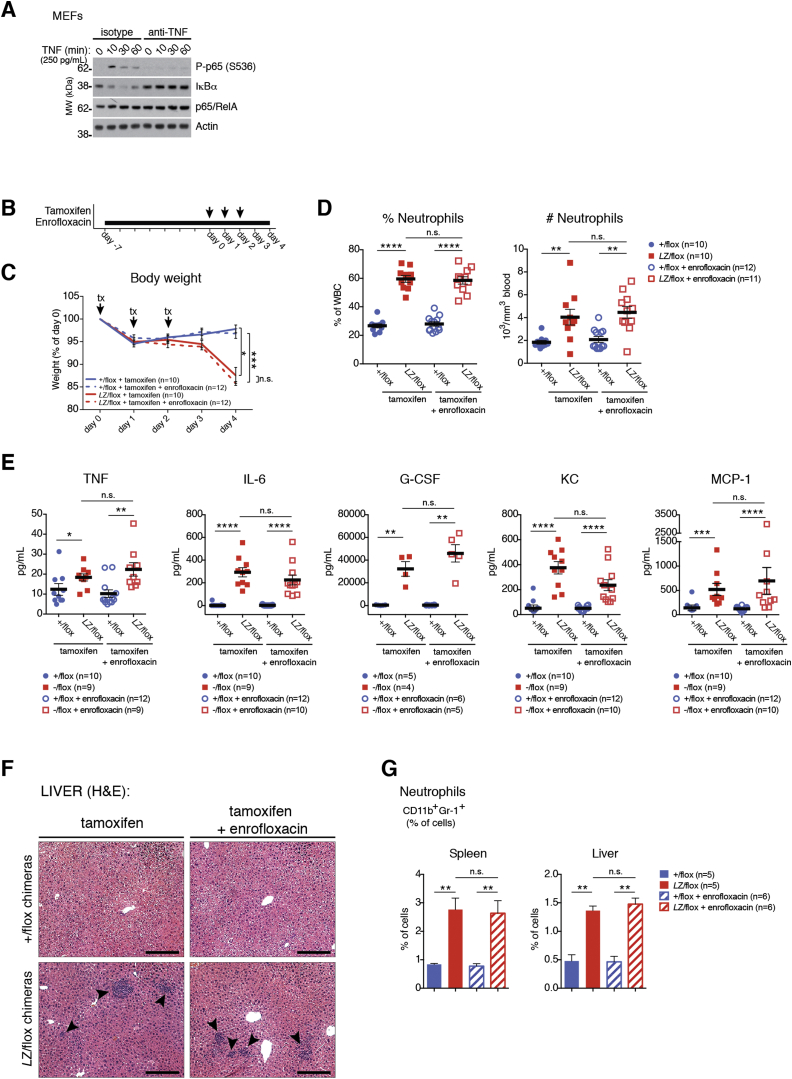
Autoactivation of BMDMs and the Effect of Antibiotics on Systemic Inflammation in OTULIN-Deficient Bone Marrow Chimeric Mice, Related to Figure 6 (A) Immunoblot showing NF-κB activation in MEFs treated with 0.25 ng/mL TNF and anti-TNF neutralizing antibodies or isotype control as indicated. (B) Schematic of the experiment indicating timing of enrofloxacin and tamoxifen treatment. (C) Enrofloxacin treatment does not rescue weight loss, indicating sterile inflammation. Tamoxifen (tx) was administered i.p. (*arrows*) to CreERT2-*Otulin*^flox^ chimeras treated with enrofloxacin or not. (D) Blood cell counts taken at day 4 from CreERT2-*Otulin*^flox^ chimeras treated with enrofloxacin or not showing that enrofloxacin does not reduce neutrophilia in CreERT2-*Otulin*^*Lac*Z/flox^ chimeras, indicating sterile inflammation. (E) Luminex multiplex analysis of cytokine and chemokine concentrations in serum from terminal bleeds on day 4 of CreERT2-*Otulin*^flox^ chimeras treated with enrofloxacin or not showing that enrofloxacin does not reduce secretion of inflammatory cytokines in CreERT2-*Otulin*^*Lac*Z/flox^ chimeras, indicating sterile inflammation. (B-E) Data were pooled from two independent experiments (except for G-CSF concentrations in E). (F) Micrographs of H&E stained sections of livers reveal inflammatory foci (*arrowheads*) in liver parenchyma showing that enrofloxacin does not reduce infiltration and inflammatory foci in CreERT2-*Otulin*^*Lac*Z/flox^ chimeras, indicating sterile inflammation. Micrographs are representative of 10 CreERT2-*Otulin*^+/flox^ and 12 CreERT2-*Otulin*^*Lac*Z/flox^ chimeras from two independent experiments. Scale bars: 200 μm. (G) Frequency of infiltrating CD11b^+^Gr-1^+^ neutrophils. (C-E and G) Data are presented as mean ± SEM, and n represents number of mice.

Finally, we examined the response of BMDMs to TNF. Since these cells secrete TNF (Figure 6E), the observed cellular response may, in part, originate from autocrine signaling. Indeed, when MEFs were treated with anti-TNF antibodies, exogenous TNF was unable to induce IκBα degradation and p65 phosphorylation (Figure S6A). Importantly, when TNF was neutralized in the culture medium of untreated BMDMs, IκBα levels decreased with similar kinetics as compared to isotype-treated BMDMs (Figure 6C, quantified in Figure 6I). This provided further strong evidence for a cell-autonomous activation of NF-κB in OTULIN-deficient BMDMs.

Autocrine signaling would be further enhanced in a positive feedback mechanism if loss of OTULIN would strengthen the response to TNF. Indeed, stimulation of BMDMs with TNF led to stronger transcriptional upregulation of *Tnf* in cells from LysMCre-*Otulin*^del/flox^ (Figure 6J), consistent with a role of OTULIN as a negative regulator in this pathway.

Together, this shows that OTULIN-deficient macrophages are unable to control LUBAC-mediated production of M1-linked polyUb chains and that this signal leads to stimulus-independent basal NF-κB activation and “sterile” inflammatory signaling, possibly enhanced by autocrine feedback. The idea of sterile inflammation is further supported in CreERT2-*Otulin*^*Lac*Z/flox^ chimeras that have been treated with broad-spectrum antibiotics to reduce the microbial load; these mice display an identical inflammatory phenotype as compared to untreated CreERT2-*Otulin*^*Lac*Z/flox^ chimeras (Figures S6B–S6G, compare Figure 2).

### Cell-Type-Specific Differences in Response to OTULIN Deficiency

While loss of OTULIN from myeloid cells and the concomitant dysregulation of M1-linked polyUb resulted in a profound inflammatory phenotype, this was not evident in mice lacking OTULIN in B or T cells (Figure 4). This is surprising since M1 signaling has recently been implicated in T cell signaling (Park et al., 2016, Redecke et al., 2016) and B cell signaling (Sasaki et al., 2013, Satpathy et al., 2015, Yang et al., 2016). The DUB-regulating M1-linked polyUb in B and T cells has not been elucidated.

**Figure 7:**
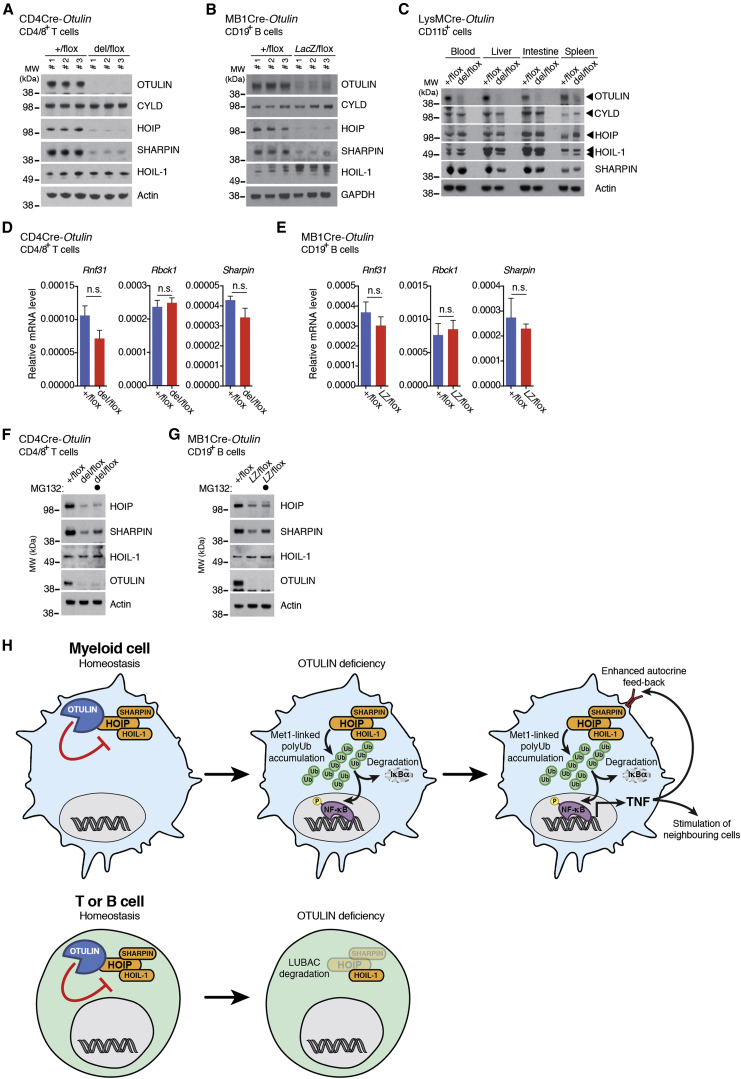
OTULIN Deficiency in T and B Cells Leads to Repression of the Linear Ubiquitin Chain Assembly Complex (A–C) Immunoblot of OTULIN, CYLD, and linear ubiquitin chain assembly (LUBAC) components in (A) splenic CD4^+^ and CD8^+^ T cells from CD4Cre-*Otulin*^flox^ mice, (B) splenic CD19^+^ B cells from MB1Cre-*Otulin*^flox^ mice, and (C) CD11b^+^ myeloid cells from multiple tissues from LysMCre-*Otulin*^flox^ mice. (D and E) Relative mRNA expression of LUBAC components from (D) splenic CD4^+^ and CD8^+^ T cells from CD4Cre-*Otulin*^flox^ mice (n = 3) and (E) splenic CD19^+^ B cells from MB1Cre-*Otulin*^flox^ mice (n = 3). Data are presented as mean ± SEM, and n represents number of biological replicas. (F and G) Immunoblot of LUBAC components in (F) splenic CD4^+^ and CD8^+^ T cells from CD4Cre-*Otulin*^flox^ mice and (G) splenic CD19^+^ B cells from MB1Cre-*Otulin*^flox^ mice treated with 10 μM MG132 proteasomal inhibitor for 6 hr as indicated. (H) Schematic showing a model of the cellular effect of OTULIN deficiency in myeloid cells and T and B cells.

To understand this, the M1-Ub signaling cascade was characterized in purified T and B cells from respective knockout mice. To our great surprise, this revealed an almost complete loss of LUBAC components HOIP and SHARPIN, but not HOIL-1, in either of the two independent models (Figures 7A and 7B). As discussed above, LysMCre-*Otulin*^*Lac*Z/flox^ BMDMs showed mildly reduced SHARPIN levels but no difference in HOIP levels. Further evaluation of myeloid cells purified from various tissues likewise showed no loss of HOIP or HOIL-1 and only marginally reduced SHARPIN levels (Figure 7C). Such tissue- and subunit-specific regulation of LUBAC components has not been observed previously.

We next investigated the mechanism of OTULIN-dependent LUBAC regulation. Transcription of HOIP (*Rnf31*), SHARPIN (*Sharpin*), and HOIL-1 (*Rbck1*) genes was unaltered in CD4Cre-*Otulin*^del/flox^ T cells and MB1Cre-*Otulin*^*Lac*Z/flox^ B cells (Figures 7D and 7E). In contrast, SHARPIN (but not HOIP) levels were partially rescued in purified T and B cells after MG132 treatment, indicating that loss of SHARPIN is due to proteasomal degradation (Figures 7F and 7G). This reveals a role for OTULIN in the stabilization of LUBAC components that warrants further investigation.

## Discussion

We provide genetic evidence that OTULIN is a crucial in vivo regulator of inflammation in mice and humans, identify TNF as a key driver of the phenotypes caused by OTULIN deficiency, and show that OTULIN deficiency and ORAS can be treated with anti-TNF/Infliximab in mice and humans.

The discovery of an OTULIN mutation in human patients with a severe inflammatory syndrome, ORAS, highlights how deregulation of a single Ub chain signal, M1-linked polyUb, causes human disease. Mutations of LUBAC components are also associated with inflammatory syndromes (Boisson et al., 2012, Boisson et al., 2015), yet these constitute loss-of-function mutations with regards to M1-polyUb signaling. In contrast, loss of function of OTULIN leads to an amplification of the M1-polyUb signal (Figure 1H). Strikingly, the net result, inflammation, is the same, highlighting how the essential M1-polyUb signal is delicately balanced to determine a cellular output.

Many DUBs have been identified to play key roles in regulating signaling pathways; however, we are unaware of another enzyme that, when removed from cells, has such global effects on a single chain type. The marked upregulation of M1-linked chains in OTULIN-deficient BMDMs and ORAS patient samples suggests that there is little redundancy for regulation of this chain type. Two recent reports implicate CYLD in the regulation of M1-polyUb signaling at receptor complexes (Draber et al., 2015, Hrdinka et al., 2016). Quantitative mass spectrometry analysis showed that OTULIN is not present at TNF and NOD2 RSCs, but while one group suggests that OTULIN’s role is restricted to LUBAC homeostasis (Draber et al., 2015), our work suggests that, while OTULIN might not stably associate with the receptor complexes, it plays active roles in receptor signaling (Hrdinka et al., 2016) (see above, Figure 6J).

Physiologically, our data support a model whereby the unrestricted accumulation of M1-linked Ub chains leads to sterile inflammation due to stimulation-independent activation of NF-κB (Figure 7H). Importantly, the role of OTULIN in NF-κB inhibition is conceptually distinct from other NF-κB-induced negative feedback regulators, such as A20 (Harhaj and Dixit, 2012). OTULIN is not under transcriptional control by NF-κB (Fiil et al., 2013, Keusekotten et al., 2013) but appears to constitutively and efficiently remove M1-linked polyUb signals. In the absence of OTULIN, LUBAC activity is unrestricted, and such signals accumulate in a deregulated fashion to initiate uncoordinated NF-κB activation and sterile inflammation. The downstream effects of this include secretion of pro-inflammatory cytokines, immune cell activation, and infiltration, culminating in severe inflammatory phenotypes (Figures 3K and 7H).

Strikingly, this is not always the case. Mice with OTULIN deficiency in B or T cells do not show overt phenotypes, and this is due to downregulation of LUBAC components HOIP and SHARPIN, but not HOIL-1, at the protein level, in OTULIN-deficient cells (Figures 7A, 7B, 7H). Genetic loss of SHARPIN in *cpdm* mice destabilizes both HOIP and HOIL-1 (Gerlach et al., 2011, Ikeda et al., 2011, Tokunaga et al., 2011), contrasting the stability of HOIL-1 in our models. This may point to differentiated use of LUBAC components in immune cells. Second, OTULIN only cleaves non-degradative M1-linked polyUb. Knockdown of OTULIN leads to M1 polyubiquitination of all three LUBAC components (Fiil et al., 2013, Hrdinka et al., 2016). It is possible that these chains, at least on HOIP and SHARPIN, are extended or edited in T and B cells by an unidentified E3 ligase to turn a non-degradative into a degradative signal. The mechanism of OTULIN-regulated LUBAC degradation requires further study, but the findings clearly suggest that whole-body OTULIN deficiency generates a complex composite phenotype, signified by cell-type-specific regulation of M1 signaling.

There are also subtle but important differences in the observed inflammatory phenotypes comparing CreERT2-*Otulin*^*Lac*Z/flox^ chimeras and LysMCre-*Otulin*^*Lac*Z/flox^ mice, which present differently in terms of severity of symptoms of disease, e.g., cachexia/weight loss. The nearly identical absolute levels of serum TNF, the key mediator of the inflammatory phenotype (Figures 1 and 3) indicates that myeloid cells are responsible for TNF production, but this does not explain the phenotypic difference. Rescue experiments suggest that G-CSF regulates emergency granulopoiesis, but not cachexia. Instead, we uncover a prominent role of IL-6, a key mediator of TNF- and inflammation-induced cachexia (Morley et al., 2006). This cytokine is higher in CreERT2-*Otulin*^*Lac*Z/flox^ chimeras as compared to LysMCre-*Otulin*^*Lac*Z/flox^ mice (Figures 2F and 4H), explaining, in part, the difference in the severity of phenotypes. Further differences include the temporal profile, with the chimera model being an induced, acute model and the myeloid-specific knockout being a constitutive, lifetime model. Finally, the numerous other upregulated cytokines in LysMCre-*Otulin*^*Lac*Z/flox^ mice, which includes anti-inflammatory IL-10, will generate a delicate balance of responses, enabling these mice to deal with inflammation induced by loss of OTULIN.

We show that excess M1-linked polyUb in cells of the immune system is harmful, but it most likely also affects other cell types and organs. For example, ORAS patients suffer from panniculitis, and it will be informative to study the role of OTULIN in skin homeostasis and inflammation. Moreover, the roles of OTULIN in embryonic development, potentially due to effects in the Wnt signaling pathway (Rivkin et al., 2013), require further investigation. Our immune-specific mouse models are not suitable to investigate this in detail. Significantly, the fact that the here-described OTULIN-related autoinflammatory syndrome/ORAS can be treated with TNF-neutralizing antibodies suggests a potential therapeutic strategy to treat conditions caused by excessive M1-linked polyUb signaling.

## STAR★Methods

### Key Resources Table

**Table:** 

REAGENT or RESOURCE	SOURCE	IDENTIFIER
**Antibodies**		

InVivoMAb anti-mouse TNF (neutralizing) (clone XT3.11)	BioXcell	Cat#BE0058; RRID: AB_1107764
InVivoMAb anti-mouse IL-6 (neutralizing) (clone MP5-20F3)	BioXcell	Cat#BE0046; RRID: AB_1107709
anti-mouse G-CSF (neutralizing) (clone 67604)	R&D Systems	Cat#MAB414; RRID: AB_2085954
InVivoMAb Rat IgG1 isotype control (clone HRPN)	BioXcell	Cat#BE0088; RRID: AB_1107775
anti-B220 conjugated to FITC (clone RA3-6B2)	eBioscience	Cat#11-0452-81; RRID: AB_465053
anti-CD3ε conjugated to FITC (clone 145-2C11)	BioLegend	Cat#17-0031-81; RRID: AB_312670
anti-CD3ε conjugated to PE-Cy7 (clone 145-2C11)	BioLegend	Cat#100319; RRID: AB_312684
anti-CD3ε conjugated to APC (clone 145-2C11)	eBioscience	Cat#17-0031; RRID: AB_469314
anti-CD4 conjugated to Alexafluor700 (clone GK1.5)	eBioscience	Cat#56-0041-82; RRID: AB_493999
anti-CD4 conjugated to Alexafluor647 (clone GK1.5)	eBioscience	Cat#50-0041-82; RRID: AB_469773
anti-CD4 conjugated to biotin (clone GK1.5)	BioLegend	Cat#100403; RRID: AB_312688
anti-CD4 conjugated to FITC (clone H129.19)	BD Biosciences	Cat#561831; RRID: AB_10892800
anti-CD4 conjugated to BrilliantViolet785 (clone RM4-5)	BioLegend	Cat#100551; RRID: AB_11218992
anti-CD5 conjugated to BrilliantViolet-510 (clone 53-7.3)	BioLegend	Cat#100627; RRID: AB_2563930
anti-CD5 conjugated to FITC (clone 53-7.3)	BD Biosciences	Cat#553020; RRID: AB_394558
anti-CD8α conjugated to FITC (clone 53-6.7)	eBioscience	Cat#11-0081-81; RRID: AB_464914
anti-CD8α conjugated to APC (clone 53-6.7)	eBioscience	Cat#17-0081-81; RRID: AB_469334
anti-CD8α conjugated to PE-Cy7 (clone 53-6.7)	eBioscience	Cat#25-0081-81; RRID: AB_469583
anti-CD8α conjugated to PE (clone 53-6.7)	eBioscience	Cat#12-0081-81; RRID: AB_465529
anti-CD8α conjugated to biotin (clone 53-6.7)	eBioscience	Cat#13-0081-81; RRID: AB_466345
anti-CD11b conjugated to PE-Cy7 (clone M1/70)	eBioscience	Cat#25-0112-81; RRID: AB_469587
anti-CD11b conjugated to eFluor-450 (clone M1/70)	eBioscience	Cat#48-0112-82; RRID: AB_1582236
anti-CD11b conjugated to BrilliantViolet-421 (clone M1/70)	BioLegend	Cat#101235; RRID: AB_10897942
anti-CD11b conjugated to FITC (clone M1/70)	BioLegend	Cat#101205; RRID: AB_312788
anti-CD11c conjugated to PE (clone N418)	BioLegend	Cat#117307; RRID: AB_313776
anti-CD11c conjugated to FITC (clone N418)	eBioscience	Cat#11-0114-81; RRID: AB_464939
anti-CD19 conjugated to FITC (clone eBio1D3)	eBioscience	Cat#11-0193-81; RRID: AB_657667
anti-CD19 conjugated to PerCP-Cy5.5 (clone eBio1D3)	eBioscience	Cat#45-0193-82; RRID: AB_1106999
anti-CD19 conjugated to Alexafluor700 (clone 6D5)	BioLegend	Cat#115528; RRID: AB_493735
anti-CD21/CD35 conjugated to APC (clone 7E9)	BioLegend	Cat#123411; RRID: AB_940395
anti-CD23 conjugated to PE-Cy7 (clone B3B4)	eBioscience	Cat#25-0232-81; RRID: AB_469603
anti-CD25 conjugated to BrilliantViolet-421 (clone PC61)	BioLegend	Cat#102033; RRID: AB_10895908
anti-CD43 conjugated to PE (clone S7)	BD Biosciences	Cat#553271; RRID: AB_394748
anti-CD44 conjugated to PE (clone IM7)	eBioscience	Cat#12-0441-81; RRID: AB_465663
anti-CD45.1 conjugated to eFluor-450 (clone A20)	eBioscience	Cat#48-0453-82; RRID: AB_1272189
anti-CD45.1 conjugated to BrilliantViolet-510 (clone A20)	BioLegend	Cat#110741; RRID: AB_2563378
anti-CD45.2 conjugated to Alexafluor700 (clone 104)	eBioscience	Cat#56-0454-81; RRID: AB_657753
and anti-CD62L conjugated to BrilliantViolet-421 (clone MEL-14)	BioLegend	Cat#104435; RRID: AB_10900082
anti-cKit/CD117 conjugated to PerCP-Cy5.5 (clone 2B8)	BioLegend	Cat#105823; RRID: AB_2131598
anti-FcεRIα conjugated to FITC (clone MAR-1)	BioLegend	Cat#134305; RRID: AB_1626102
anti-IgD conjugated to BrilliantViolet-650 (clone 11-26c.2a)	BioLegend	Cat#405721; RRID: AB_2562731
anti-IgM conjugated to PerCP-Cy5.5 (clone RMM-1)	BioLegend	Cat#406511; RRID: AB_2075944
anti-Ly6G/Gr-1 conjugated to FITC (clone RB6-8C5)	eBioscience	Cat#11-5931-81; RRID: AB_465313
anti-Ly6G/Gr-1 conjugated to PE (clone RB6-8C5)	eBioscience	Cat#12-5931-81; RRID: AB_466044
anti-Ly6G/Gr-1 conjugated to APC (clone RB6-8C5)	eBioscience	Cat#17-5931-81; RRID: AB_469475
anti-NK1.1 conjugated to BrilliantViolet-421 (clone PK136)	BioLegend	Cat#108731; RRID: AB_10895916
anti-NK1.1 conjugated to FITC (clone PK136)	BioLegend	Cat#108705; RRID: AB_313392
anti-Sca1 conjugated to PE-Cy7 (clone D7)	eBioscience	Cat#25-5981-81; RRID: AB_469668
anti-Ter-119 conjugated to FITC (clone TER-119)	eBioscience	Cat#11-5921; RRID: AB_2206887
anti-A20	Cell Signaling Technology	Cat#4625; RRID: AB_2204524
anti-Actin (clone C4)	Millipore	Cat#MAB1501R; RRID: AB_94235
anti-CYLD	Santa Cruz Biotechnology	Cat#sc-74435; RRID: AB_1122022
anti-GAPDH	Ambion	Cat#AM4300; RRID: AB_437392
anti-HOIL-1/RBCK1	Novus Biologicals	Cat#NBP2-27105; RRID: AB_2576210
anti-HOIL-1/RBCK1	Santa Cruz Biotechnology	Cat#sc-49718; RRID: AB_2175281
anti-HOIP/RNF31	Abcam	Cat#46322; RRID: AB_945269
anti-mouse HOIP/RNF31	Laboratory of Kazuhiro Iwai	Tokunaga et al., 2011; RRID: N/A
anti-IκBα	Cell Signaling Technology	Cat#9242; RRID: AB_10694550
anti-IRF-3	Santa Cruz Biotechnology	Cat#sc-9082; RRID: AB_2264929
anti-phospho-IRF-3 (pS396)	Cell Signaling Technology	Cat#4947; RRID: AB_823547
anti-Lys63-linked ubiquitin (clone Apu3)	Millipore	Cat#05-1308; RRID: AB_1587580
anti-Lys48-linked ubiquitin (clone Apu2)	Millipore	Cat#05-1307; RRID: AB_1587578
anti-Met1-linked/linear ubiquitin (clone LUB9)	LifeSensors	Cat#AB130; RRID: AB_2576211
anti-Met1-linked/linear ubiquitin (clone 1E3)	Millipore	Cat#MABS199; RRID: AB_2576212
anti-NIK	Santa Cruz Biotechnology	Cat#sc-8417; RRID: AB_628021
anti-NEMO/IKKγ	Santa Cruz Biotechnology	Cat#sc-8330; RRID: AB_2124846
anti-OTULIN	Cell Signaling Technology	Cat#14127; RRID: AB_2576213
anti-p38 (clone M138)	Abcam	Cat#ab31828; RRID: AB_881839
anti-phospho-p38 (pT180/pY182) (clone ERP18120)	Abcam	Cat#ab195049; RRID: AB_2576214
anti-p65/RelA	Cell Signaling Technology	Cat#8242; RRID: AB_10860244
anti-phospho-p65/RelA (pS563) (clone 93H1)	Cell Signaling Technology	Cat#3033; RRID: AB_331284
anti-SHARPIN	Proteintech	Cat#14626-1-AP; RRID: AB_2187734
anti-ubiquitin (clone UBI-1)	Novus Biologicals	Cat#NB300-130; RRID: AB_2238517
anti-mouse CD19-coupled MACS MicroBeads	Miltenyi Biotec	Cat#130-052-201; RRID: N/A
anti-mouse CD11b-couple MACS MicroBeads	Miltenyi Biotec	Cat#130-049-601; RRID: N/A
anti-Biotin-coupled MACS MicroBeads	Miltenyi Biotec	Cat#130-090-485; RRID: N/A

**Chemicals, Peptides, and Recombinant Proteins**		

Recombinant mouse M-CSF	R&D Systems	Cat#416-ML-050
Recombinant mouse TNF	GIBCO	Cat#PMC3014
Ultrapure LPS from *E. coli* K12	InvivoGen	Cat#tlrl-peklps
Staurosporine from *Streptomyces sp.*	Sigma-Aldrich	Cat#S6942; CAS 62996-74-1
MG132 (Z-Leu-Leu-Leu-al)	Sigma-Aldrich	Cat#C2211; CAS 133407-82-6
Tamoxifen	Sigma	Cat#T5648-1G; CAS 10540-29-1
3-(4,5-dimethylthiazol-2-yl)-2,5-diphenyltetrazoliym bromide (MTT)	Sigma	Cat#M2128-250MG; CAS 298-93-1
Collagenase D	Roche	Cat#11088866001
Liberase	Roche	Cat#5401020001
DNase I	Sigma-Aldrich	Cat#D5025
Percoll PLUS	GE Healthcare	Cat#17-5445-02
Recombinant OTULIN-WT	Laboratory of David Komander	Keusekotten et al., 2013
Recombinant OTULIN-C129A/L272P	This paper	N/A

**Critical Commercial Assays**		

MILLIPLEX MAP Cytokine/Chemokune Magnetic Bead Panel – Premixed 25-plex	Merck Millipore	Cat#MCYTOMAG-70K-PMX
Mouse BAFF/BLyS/TNFSF13B Quantikine ELISA kit	R&D Systems	Cat#MBLYS0
Mouse G-CSF Quantikine ELISA kit	R&D Systems	Cat#MCS00
Mouse anti-nuclear antigen/ENA IgA+G+M ELISA kit	Alpha Diagnostics	Cat#5210
Mouse anti-Smith antigen IgA+G+M ELISA kit	Alpha Diagnostics	Cat#5405
Mouse anti-dsDNA IgA+G+M ELISA kit	Alpha Diagnostics	Cat#5110
Mouse Total IgG Ready-Set-Go ELISA kit	eBioscience	Cat#88-50400-22
IgG Mouse ELISA kit	Abcam	Cat#ab151276
Mouse Isotyping Panel 1 multiplex sandwich electrochemiluminescence immunoassay kit	Meso Scale Discovery	Cat#K15183-1
MACS Dead Cell Removal Kit	Miltenyi Biotec	Cat#130-090-101

**Experimental Models: Organisms/Strains**		

Mouse: *ACTB*-FLPe	Laboratory of Susan M. Dymeki	Rodríguez et al., 2000
Mouse: *ROSA26-*CreERT2	Laboratory of Ernesto Bockamp	Hameyer et al., 2007
Mouse: *LysM-*Cre	Laboratory of Irmgard Förster	Clausen et al., 1999
Mouse: *Mb1*-Cre	Laboratory of Michael Reth	Hobeika et al., 2006
Mouse: *Cd4*-Cre	Laboratory of Christopher B. Wilson	Lee et al., 2001

**Experimental Models: Cell Lines**		

*Otulin/Fam105b*-targeted JM8A3.N1 ES cells	EuMMCR	*Fam105b*^tm1a(EUCOMM)Hmgu^
HEK293	N/A	N/A

**Recombinant DNA**		

pEGFP-N1-OTULIN-WT	Laboratory of David Komander	Keusekotten et al., 2013.
pEGFP-N1-OTULIN-L272P	This paper	N/A

**Sequence-Based Reagents**		

For primer sequences, please see Table S8.		

**Software and Algorithms**		

HomozygosityMapper	Seelow et al., 2009	http://www.homozygositymapper.org/
Annovar / wAnnovar	Yang and Wang, 2015	http://wannovar.usc.edu/index.php
SAMtools	Li et al., 2009	http://samtools.sourceforge.net/

**Other**		

Exome Variant Server	NHLBI GO Exome Sequencing Project (ESP)	http://evs.gs.washington.edu/EVS/
EXaC Browser	Exome Aggregation Consortium	http://exac.broadinstitute.org/gene/ENSG00000154124

### Contact for Reagent and Resource Sharing

Further information and requests for reagents may be directed to, and will be fulfilled by the corresponding author David Komander (dk@mrc-lmb.cam.ac.uk).

### Experimental Models and Subject Details

#### ORAS Patients

##### Consent Information

Written informed consent was obtained for all subjects and family members (n = 7). The study was approved by the South Birmingham Research Ethics Committee and performed in accordance with the ethical standards laid down in the 1964 Declaration of Helsinki.

#### Clinical Description of ORAS Patients, Related to Figure 1 and Tables S1, S2, and S3

##### Patient IV:3

The consultand is the first child born to consanguineous parents (III:3 and III:4) (Figure 1A) at 34 weeks gestation via normal vaginal delivery and was small for gestational age. She presented at 3 weeks of age with protracted diarrhea and failure to thrive. She required total parenteral nutrition (TPN) and later nasogastric tube feeds. During her lengthy admission she was noted to have recurrent episodes of a widespread nodular erythematous rash associated with fever, elevated white blood cell count, neutrophilia, raised C-reactive protein (CRP) (Figures 1B-D), and an exacerbation of her diarrhea. An initial skin biopsy, taken as the rash was resolving, showed non-specific changes. Duodenal biopsies showed microvillous dystrophy. She also had mild hepatomegaly. A liver biopsy showed TPN-associated liver disease with micronodular cirrhosis and macrovisicular steatosis. Liver function later returned to normal, although hepatomegaly and the permanent damage from cirrhosis persisted. There are no records of splenomegaly in this patient. Echocardiogram identified an atrial septal defect.

The patient was extensively investigated to identify the cause of her protracted diarrhea and episodic skin rash including a number of immunological investigations. Of note she had normal alpha 1 anti-trypsin level, normal functional antibodies, normal lymphocyte subsets although with a modest increase in CD19^+^ B cells (2120 cells/mm^3^; normal 500-1500), normal lymphocyte function tests, neutrophil function test, and normal NBT tests. She did have elevated IgG, IgM, and IgA levels (Table S1), raised CRP (Figure 1C), elevated C1q (171 mg/L; normal 80-150 mg/L) and C3 (2.75 g/L; normal 0.75-1.75 g/L), and she was positive for anti-neutrophil cytoplasmic antibody (ANCA) and anti-smooth muscle antibody (SMA), both 1:100 titers. When she was treated with intravenous methylprednisolone, her fever, diarrhea, and rash would resolve, but these clinical features would return when attempts were made to change to oral prednisolone. Azathioprine and methotrexate were also trialed. In a course of 3 months she had three cases of severe bilateral pneumonia progressing to sepsis and renal tubular necrosis, hematuria, proteinuria, and raised urea and creatinine requiring admittance to the pediatric intensive care unit. She survived two episodes, but sadly died at 16 months during the third episode of pneumococcal septicemia, which led to respiratory collapse.

##### Patient IV:4

The consultand is the second child born to consanguineous parents (III:3 and III:4) (Figure 1A) at 36 weeks gestation via normal vaginal delivery with a birth weight of 2 kg. She was diagnosed with relapsing nodular panniculitis at 3 days of age. A skin biopsy showed no evidence of vasculitis. This rash, similarly to her sister (patient IV:3) and cousin (patient V:2), was episodic and associated with diarrhea (bloody at presentation), vomiting, fever, and painful swollen joints and difficulty sleeping. She had elevated white blood cell counts with pronounced neutrophilia as well as raised CRP levels (Figures 1B-D), as well as elevated C3 (2.77 g/L; normal 0.75-1.75 g/L), but was negative for ANCA autoantibodies (she was not tested for other autoantibodies). She had normal lymphocyte subsets. There are no records of hepatosplenomegaly in this patient. She required nasogastric tube feeds for failure to thrive. All growth parameters were below the 0.4th centile. She also had bilateral cataracts diagnosed at the age of 5 months and developmental delay. The cataracts were not present in the neonatal period on formal ophthalmological examination and were not secondary to steroid use.

She was treated with prednisolone from the age of 1 month, and with azathioprine and Anakinra (recombinant IL-1R-antagonist) unsuccessfully. She had recurrent urinary tract infections and viral illnesses, which coincided with immunosuppressive therapy. During a severe episode of inflammation at the age of five she demonstrated features consistent with a high cell turnover including elevated potassium, phosphate, uric acid, LDH, elevated white blood cell count and a metabolic acidosis. She was admitted to pediatric intensive care with acute renal failure, pulmonary edema and an ileus, but sadly passed away just before turning 5 years old. Treatment had included intubation and ventilation, intravenous methylprednisolone, dexamethasone, and methotrexate. In this patient, Familial Mediterranean Fever, Chronic Infantile Neurological Cutaneous Articular syndrome, Tumor necrosis factor Receptor-Associated Periodic Syndrome, and Hyper IgD syndrome had all been excluded by genetic analysis.

##### Patient V:2

The consultand is the second child born prematurely at 28+6 weeks gestation to consanguineous parents (IV:1 and IV:2) (Figure 1A) with a birth weight of 1.23 kg. He developed relapsing nodular panniculitis at 8 weeks of age while he was in the neonatal intensive care unit. Prior to the appearance of his skin rash, he had repeated episodes of possible infection with raised CRP levels and elevated white blood cell count and neutrophilia (Figures 1B-D), but no focus of infection could be identified. The rash was biopsied and this confirmed inflammation in the dermis extending into the subcutaneous layer with a mixed inflammatory cell infiltrate. No granulomas or vasculitis was seen. He has had frequent flare-ups involving widespread painful lumps in the skin lasting 2 days to 2 weeks. During these episodes he was systemically unwell with fever, vomiting, diarrhea (sometimes bloody), inflamed painful joints, swollen feet, and weight loss associated with elevated CRP levels and white blood cell count (Figures 1C and 1D), with a neutrophilia showing toxic granulation and a left shift. He would lose his appetite, lose weight, and become dehydrated, which required admission to the high dependency unit on some occasions. These episodes appeared to resolve with an increase in the dose of prednisolone. In addition, he seemed susceptible to frequent infections especially viral illnesses including varicella zoster virus (VZV), influenza A, respiratory syncytial virus (RSV), adenovirus, and cytomegalovirus (CMV), although these coincided with immunosuppressive therapy. He had poor weight gain, slow linear growth, developmental delay, mild learning difficulties, congenital hydroceles, dental caries, a pathological osteoporotic tibial fracture and he developed juvenile cortical cataracts at 2-3 years of age, which were treated with a bilateral lensectomy and vitrectomy. There are no records of hepatosplenomegaly in this patient.

On examination, his growth parameters were all less than the 0.4th centile. He had a prominent nodular rash in keeping with a flare of panniculitis. He had coarse hair with bushy eyebrows, slight hyper-telorism, broad nasal bridge, prominent nose, protruding normally formed ears and a prominent chin.

He had comprehensive immunological investigations which were normal including lymphocyte subset panel and proliferation analysis, a normal neutrophil oxidative burst, and normal expression of CD11a,b,c, CD18, CD55, CD95, MHCI, MHCII, and CD25. He had increased IgA and IgM levels (Table S1) in serum and a single strongly positive anti-smooth muscle antibody (SMA; 1:320 titer), but no other autoantibodies. He does not have alpha-1 antitrypsin deficiency or pancreatitis. The most recent skin biopsy demonstrated inflammatory infiltrates composed of lymphocytes and neutrophils within the subcutis, associated with foci of fat necrosis where neutrophils are particularly prominent. The inflammation seemed predominantly septal, but with extension into the lobules. Some of the septal blood vessels showed edema of their walls, but no frank vasculitis was identified. The features were very similar to those seen in the biopsy from his cousin (patient IV:4) and suggested a neutrophil-rich panniculitis with fat necrosis favoring septal distribution.

He has been treated both with systemic steroids and Anakinra (recombinant IL-1R-antagonist). Neither medication successfully prevented the exacerbations or additional symptoms. Infliximab (TNF neutralizing antibody) was introduced eight years ago (at age ∼3) and has successfully controlled the disease. He has had a couple of minor exacerbations when the frequency of the Infliximab treatments was decreased, or the dose per kg fell to nearly 5 mg/kg. In addition to this, he takes prophylactic methotrexate, azithromycin, and aciclovir.

#### Mice

All animal experiments were undertaken with the approval of the UK Home Office. All mice were on a C57BL/6 or B6.SJL background and maintained under specific pathogen-free conditions in individually ventilated cages (Techniplast GM500, Techniplast) on Lignocel FS14 spruce bedding (IPS, Ltd.) with environmental enrichment (fun tunnel, chew stick, and Enviro-Dri nesting material (LBS)) at 19-23°C with light from 7.00 a.m. to 7.00 p.m. and fed Dietex CRM pellets (Special Diet Services) ad libitum.

*Otulin/Fam105b*-targeted JM8A3.N1 (C57BL/6 background strain) ES cells (*Fam105b*^tm1a(EUCOMM)Hmgu^) were obtained from EUCOMM and used to generate mice bearing neomycin selection and *Lac*Z cassettes and a *loxP*-flanked exon 3 of *Otulin,* termed the targeted “*Lac*Z“ or “*L*Z” (figures) allele (see also Figure S1). Heterozygous *Otulin*^+/*Lac*Z^ mice were normal and asymptomatic (data not shown), but intercrossing of *Otulin*^+/*Lac*Z^ mice failed to generate viable *Otulin*^*Lac*Z/*Lac*Z^ offspring, consistent with previous work (Bonizzi and Karin, 2004, Rivkin et al., 2013). The neomycin selection and *Lac*Z cassettes were removed from the targeted allele by intercrossing with FLPe-recombinase expressing mice (Rodríguez et al., 2000) to generate the conditional, floxed allele, which can be conditionally ablated by the Cre recombinase leading to a frameshift and a premature stop codon (*p.Glu77Glyfs^∗^Ter3*) (Figures S2A and S2B), and the *Otulin*^*flox*^ strain (*Otulin*^+/flox^ and *Otulin*^*Lac*Z/flox^ mice). *Otulin*^flox^ mice were bred with *Rosa26*-Cre-*ERT2* (Hameyer et al., 2007) (CreERT2-*Otulin*^flox^), *LysM*-Cre (Clausen et al., 1999) (LysMCre-*Otulin*^flox^), *CD4*-Cre (Lee et al., 2001) (CD4Cre-*Otulin*^flox^), and *Mb1*-Cre (Hobeika et al., 2006) (MB1Cre-*Otulin*^flox^) mice to facilitate conditional deletion of *Otulin*. Pilot experiments showed that CreERT2-*Otulin*^*Lac*Z/flox^ mice reacted adversely to tamoxifen administration, becoming moribund within a day of *Otulin* deletion (data not shown).

In individual experiments, mice were matched for age and background strain. All experiments, except for embryo staining (see below) were performed on adult mice. For ERT2Cre-*Otulin*^flox^ bone marrow chimeric mice, recipient mice were 2-3 months old and in each experiment were either all male or all female. No differences in results were observed between experiments performed on male and female mice. For LysMCre-*Otulin*^flox^, CD4Cre-*Otulin*^flox^, MB1Cre-*Otulin*^flox^ mice, experiments were performed with a mix of male and female mice in experimental groups. LysMCre-*Otulin*^flox^, CD4Cre-*Otulin*^flox^, MB1Cre-*Otulin*^flox^ mice used in experiments were 2-9 months old. Mice were allocated to experimental groups based on genotype. Grouping of mice into cages was determined at weaning, but where possible animals of equivalent age and gender were allocated to each experimental group. Where multiple groups of the same genotype were required, these were allocated randomly to the particular treatment conditions. Sample sizes were estimated using pilot experiments.

#### Bone Marrow-Derived Macrophages

Bone marrow-derived macrophages (BMDMs) were generated from bone marrow cells derived from tibias, femurs, and pelvic bones from LysMCre-*Otulin*^+/flox^, LysMCre-*Otulin*^*Lac*Z/flox^ or LysMCre-*Otulin*^del/flox^ mice. Bones were flushed in PBS supplemented with 3% (v/v) FCS and cells were cultured and differentiated in RPMI 1640 + GlutaMAX supplemented with 10% (v/v) FCS, Penicillin/Streptomycin, 5 μM β-mercapto ethanol, nonessential amino acids (GIBCO), and 20 ng/mL recombinant mouse M-CSF (R&D Systems, Minneapolis, MN) as described previously (Damgaard et al., 2012). BMDMs denoted ‘untreated’ in experiments indicates no exogenous stimulation of these cells after differentiation.

#### Cell Lines

HEK293 cells and mouse embryonic fibroblasts (MEFs) were cultured in DMEM + GlutaMAX supplemented with 10% (v/v) FCS and Penicillin/Streptomycin at 37°C in a humidified atmosphere at 5% CO_2_ unless otherwise indicated in figure legends or method details.

### Method Details

#### Molecular Genetic Analysis

Blood genomic DNA was isolated using the DNeasy kit (QIAGEN). A genome-wide linkage scan was carried out using the Affymetrix 250K SNP microarray with DNA from the three affected patients (V:2, IV:3 and IV:4). Homozygous regions were identified in affected patients using the program HomozygosityMapper (http://www.homozygositymapper.org/) (Seelow et al., 2009) and further analyzed to confirm or refute linkage by typing microsatellite markers in all family members from whom DNA was available. Direct sequencing of the genes within the identified region was prioritised according to putative function and position. The genomic DNA sequence of candidate genes was taken from Ensembl (http://www.ensembl.org/index.html) and primer pairs for the translated exons were designed using ExonPrimer software (https://ihg.gsf.de/ihg/ExonPrimer.html). Individual exons and flanking sequences were amplified using standard polymerase chain reaction (PCR) (primer details and conditions on request). PCR products were directly sequenced by the Big Dye Terminator Cycle Sequencing kit and run on an ABI PRISM 3730 DNA Analyzer (Applied Biosystem). Sanger sequencing was repeated on at least two independent PCR products to confirm sequence variants. DNA sequences were analyzed using the Chromas software (Technelysium). Whole Exome Sequencing was performed as described previously (Clark et al., 2014). Exon capture was performed with the SureSelect All Exon 50Mb Target Enrichment System (Agilent) and massively parallel DNA sequencing was undertaken on an Illumina AnalyserIIx with 76bp paired end reads. Single nucleotide substitutions and small insertion deletions were detected and quality filtered within the SamTools software package (http://samtools.sourceforge.net/) (Li et al., 2009) and in-house software tools. Variants were annotated with the Annovar tool (http://wannovar.usc.edu/index.php) (Yang and Wang, 2015). Filtering of variants for novelty was performed by comparison to dbSNP132 and 1000 Genomes SNP calls (June 2011) and patient variants were compared to variants identified in 250 control exomes sequenced and analyzed in a similar manner. The Exome Variant Server (http://evs.gs.washington.edu/EVS/) and the EXaC Browser (http://exac.broadinstitute.org/gene/ENSG00000154124) datasets were accessed October 2015.

#### β-Galactosidase Staining of Mouse Embryos

E13.5 *Otulin*^+/*Lac*Z^ and *Otulin*^+/+^ embryos were dissected from the uterine tracts and immediately transferred to ice cold 4% PFA in PBS and left to fix for 1 hr. Fixed embryos were washed in rinse buffer (5 mM EGTA, 0.01% (w/v) deoxycholate, 0.02% NP-40 (v/v), 2 mM MgCl_2_ in PBS) and transferred to staining buffer (5 mM K_3_[Fe(CN)_6_], 5 mM K_4_[Fe(CN)_6_], 5 mM EGTA, 0.01% deoxycholate (w/v), 0.02% NP-40 (v/v), 2 mM MgCl_2_, 1 mg/mL 5-bromo-4-chloro-3-indolyl-β-D-galactopyranoside (X-gal) in PBS) and left to incubate 24 hr in the dark at 37°C. After staining, embryos were washed in rinse buffer and post-fixed in 4% PFA in PBS for 48 hr at room temperature. Embryos were then washed in PBS and dehydrated by sequential washes in increasing concentrations of ethanol ending with 100% ethanol for 30 min and then cleared in methyl salicylate (Sigma Aldrich) for 30 min. Embryos were allocated to experimental groups based on genotype. This experiment was performed on embryos from two independent litters, and a total of five *Otulin*^+/+^ and five *Otulin*^+/*Lac*Z^ embryos were analyzed.

#### Mixed Bone Marrow Chimeras

One week before transplantation, the drinking water of recipient mice was supplemented with 0.1 mg/mL enrofloxacin (Baytril®, Bayer). Bone marrow cells (2 × 10^6^) from wild-type CD45.1^+^ B6/SJL mice and CD45.2^+^ CreERT2-*Otulin*^+/flox^ or CreERT2-*Otulin*^*Lac*Z/flox^ mice were resuspended in 100 μL PBS at a 1:1 ratio and then injected intravenously (i.v.) into 2-3 months old sex- and age-matched γ-irradiated C57BL/6 recipients (given two doses of 4.5 Gy). At 6-8 weeks after reconstitution, mice were given three doses of tamoxifen (Sigma-Aldrich, St Louis, MO; 1 mg in sunflower oil with 10% ethanol per dose) intraperitoneally (i.p.). Mice were closely monitored and weighed daily. At the onset of weight loss (3-6 days after initial tamoxifen dose) mice were culled and samples taken for analyses. Mixed bone marrow chimeric mice were allocated to experimental groups based on genotype of the transplanted bone marrow. Where multiple groups of the same genotype were required, these were allocated randomly to the particular treatment conditions.

#### In Vivo Cytokine Neutralization

For in vivo neutralization of cytokines, mixed bone marrow chimeric mice (described above) were injected i.v. with antibodies two to four hours before tamoxifen injection. Chimeric mice were injected with 1 mg/mouse *InVivo*Mab anti-mouse TNF (clone XT3.11, BE0058, BioXcell, West Lebanon, NH), 1 mg/mouse *InVivo*Mab anti-mouse IL-6 (clone MP5-20F3, BE0046, BioXCell), 250 μg/mouse anti-mouse G-CSF (clone 67604, MAB414, R&D Systems), or the equivalent amount of *InVivo*Mab Rat IgG1 isotype control (clone HRPN, BE0088, BioXCell). Mixed bone marrow chimeric mice were allocated to the experimental groups based on genotype of the transplanted bone marrow. Where multiple groups of the same genotype were required, these were allocated randomly to the particular treatment conditions.

#### Cytokine/Chemokine, Autoantibody, and Immunoglobulin Analysis

Cytokine/chemokine multiplex analysis was carried out using Luminex xMAP technology. Serum and medium samples were analyzed using magnetic MILLIPLEX MAP antibody-conjugated beads (Merck-Millipore, Bedford, MA) according to the manufacturer’s instructions on a Luminex MAGPIX instrument with the xPONENT 4.2 software. For some G-CSF measurements, samples were diluted 1/10. For cytokine measurements from BMDMs, cells were split and equal numbers were reseeded in fresh medium 24 hr prior to sample collection. BAFF levels, and G-CSF levels in some experiments, were determined using the Mouse BAFF/BLyS/TNFSF13B Quantikine ELISA or the Mouse G-CSF Quantikine ELISA kits (R&D Systems), respectively, with a 1/20 dilution of samples for BAFF and 1/10 for G-CSF according to the manufacturer’s instructions. Autoantibodies were detected using mouse anti-nuclear antigen/ENA IgA+G+M, mouse anti-Smith antigen IgA+G+M, and mouse anti-dsDNA IgA+G+M ELISA kits (Alpha Diagnostics, San Antonio, TX) with 1/100 dilution of samples according to the manufacturers instructions. All samples were run in duplicate. Serum total IgG levels were determined using the Mouse Total IgG Ready-Set-Go ELISA kit (eBioscience, San Diego, CA) or the IgG Mouse ELISA kit (Abcam, Cambridge, UK) according to the manufacturer’s instructions. Serum immunoglobulin isotyping was performed using the Mouse Isotyping Panel 1 multiplex sandwich electrochemiluminescence immunoassay kit (Meso Scale Discovery, Gaithersburgh, MD) with 1/100,000 dilution of samples according to the manufacturer’s instructions. All samples were run in duplicate. Patient C-reactive protein (CRP) were analyzed on an Abbott Archetect C1600.

#### Histology

Tissue samples were harvested in 3% (v/v) FCS in PBS on ice, transferred to 10% neutral buffered formalin, and fixed for 48 hr at room temperature. Fixed tissues were sent to AML Laboratories, Inc., Baltimore, MD, for paraffin embedding, sectioning, and Hematoxylin & Eosin or Masson’s Trichrome staining. Micrographs were taken on a Carl Zeiss Axioplan microscope with an Axiocam camera and processed using the Fiji software (Schindelin et al., 2012). Scale bars represent 200 μm.

#### Flow Cytometry and Cell Sorting

Single cells in a solution of 2% (v/v) FCS in PBS at 4°C were then blocked with anti-CD32 antibodies (clone 2.4G2; BioXCell, West Lebanon, NH), washed, and then incubated with fluorophore-conjugated antibodies. Fixable Viability Dye eFluor780 (eBioscience, San Diego, CA) was included in all analyses to exclude dead cells. Cells were analyzed on a BD Fortessa (BD Bioscience, Oxford, United Kingdom) or were sorted with a MoFlo Synergy cell sorter (Beckman Coulter, Inc., Fullerton, CA) according to the manufacturers’ standard operating procedures. Data were analyzed with FlowJo software version X.07. For quantification of cells numbers of infiltrating cells, the absolute number of cells in a gate was multiplied by the dilution factor of the sample and corrected for the percentage of dead cells. Fluorophore-coupled antibodies were anti-CD4 conjugated to Alexafluor700 or Alexafluor647 (clone GK1.5, eBioscience), anti-CD4 conjugated to FITC (clone H129.19, BD Pharmigen), anti-CD4 conjugated to BrilliantViolet785 (clone RM4-5, BioLegend), anti-CD8α conjugated to FITC, APC, PE-Cy7 or PE (clone 53-6.7, eBioscience), anti-CD11b conjugated to PE-Cy7 or eFluor-450 (clone M1/70, eBioscience), anti-CD11b conjugated to BrilliantViolet-421 or FITC (clone M1/70, BioLegend), anti-CD11c conjugated to PE (clone N418, BioLegend), anti-CD11c conjugated to FITC (clone N418, eBioscience), anti-CD19 conjugated to FITC or PerCP-Cy5.5 (clone 1D3, eBioscience), anti-CD19 conjugated to Alexa Fluor-700 (clone 6D5, BioLegend), anti-CD45.1 conjugated to eFluor-450 (clone A20, eBioscience), anti-CD45.1 conjugated to BrilliantViolet-510 (clone A20, BioLegend), anti-CD45.2 conjugated to Alexa Fluor-700 (clone 104, eBioscience), anti-Ly6G/Gr-1 conjugated to FITC, PE, or APC (clone RB6-8C5, eBioscience), anti-NK1.1 conjugated to BrilliantViolet-421 or FITC (clone PK136, BioLegend), anti-Ter-119 conjugated to FITC (clone TER-119, eBioscience), anti-FcεRI conjugated to FITC (clone MAR-1, BioLegend), anti-CD43 conjugated to PE (clone S7, BD Pharmigen), anti-CD25 conjugated to BrilliantViolet-421 (clone PC61, BioLegend), anti-CD23 conjugated to PE-Cy7 (clone B3B4, eBioscience), anti-CD21 conjugated to APC (clone 7E9, BioLegend), anti-IgM conjugated to PerCP-Cy5.5 (clone RMM-1, BioLegend), anti-IgD conjugated to BrilliantViolet-650 (clone 11-26c.2a, BioLegend), anti-CD5 conjugated to BrilliantViolet-510 (clone 53-7.3, BioLegend), anti-Sca1 conjugated to PE-Cy7 (clone D7, eBioscience), anti-cKit/CD117 conjugated to PerCP-Cy5.5 (clone 2B8, BioLegend), anti-B220 conjugated to FITC (clone RA3-6B2, eBioscience), anti-CD3 conjugated to FITC or PE-Cy7 (clone 145-2C11, BioLegend), anti-CD3 conjugated to APC (clone 145-2C11, eBioscience), anti-CD5 conjugated to FITC (clone 53-7.3, BD Pharmigen), anti-CD44 conjugated to PE (clone IM7, eBioscience), and anti-CD62L conjugated to BrilliantViolet-421 (clone MEL-14, BioLegend).

Lineage (Lin) stain panel consisted of anti-B220, anti-CD19, anti-CD3, anti-CD4, anti-CD5, anti-CD8, anti-CD11b, anti-CD11c, anti-NK1.1, anti-FcεRI, and anti-Ter119 antibodies conjugated to FITC.

#### Bone Marrow-Derived Macrophages

Bone marrow-derived macrophages (BMDMs) were considered “untreated” when no exogenous stimulation was given after completed differentiation (day 7). For assessment of IκBα stability, BMDMs were washed thoroughly in PBS and then incubated with 10 μg/mL anti-TNF neutralizing antibodies (clone XT3.11, BE0058, BioXCell) or isotype control (clone HPRN, BE0088, BioXcell) in complete RPMI for 30 min before they were treated with 50 μg/mL cycloheximide in DMSO (CHX; Santa Cruz Biotechnology) for the indicated times. To verify TNF neutralization, immortalized mouse embryonic fibroblasts (MEFs) were cultured in medium containing the TNF neutralizing antibody or the isotype control and stimulated with 0.25 ng/mL recombinant mouse TNF (GIBCO) for the indicated times. For signaling experiments, BMDMs were stimulated with 1 ng/mL recombinant mouse TNF (GIBCO) for the indicated times before they were lysed for quantitative RT-PCR analysis.

#### Tissue Preparation and MACS Cell Separation

Single cell suspensions were obtained by gentle mechanical disruption of tissues using a syringe plunger and a 70 μm cell strainer. Lungs were digested for 30 min with collagenase D (Roche; 0.7 mg/mL in 2% (v/v) FCS in PBS) before disruption. For CD11b^+^ cell isolation from livers for immunoblotting (Figure 7C), mashed livers were resuspended in 44% Percoll PLUS (GE Healthcare) in PBS and centrifuged at 2000 rpm for 20 min with low deceleration. The top layer of hepatocytes and Percoll were then aspirated, and the pelleted leukocytes were resuspended in 4.5 mL ice-cold PBS, vortexed for 15 s, and then added 500 μL 10x PBS and vortexed again. The remaining leukocytes were pelleted for centrifugation and subjected to isolation using MACS MicroBeads (see below). For lamina propria leukocyte preparations from small intestine and colon (for Figure 7C), the mesentery was removed, the intestinal content gently squeezed out, and the intestines were washed in PBS/HEPES (PBS + 10 mM HEPES) and then cut into 2-3 cm pieces. Digestion and cell isolation was then performed using 0.06 mg/mL Liberase (Roche) and 60 μg/mL DNase I (Sigma Aldrich) as previously described (Morrison et al., 2013). Blood leukocytes were prepared from whole EDTA-blood by diluting 500 μL blood in 8.5 mL ice-cold distilled water and vortexing for 15 s. Immediately thereafter 1 mL 10x PBS was added the preparation was vortexed for 10 s and the blood leukocytes were pelleted by centrifugation. The remaining leukocytes were pelleted for centrifugation and subjected to isolation using MACS MicroBeads (see below). All cell preparations and suspensions were passed through 70 μm nylon cell strainers again before cell isolation. CD19^+^ B cells were isolated from spleen preparations using anti-mouse CD19-coated magnetic MACS MicroBeads (Miltenyi Biotec, Bergisch Gladbach, Germany), CD4^+^ and CD8^+^ T cells were isolated from spleen preparations using anti-biotin-coated magnetic MACS MicroBeads (Miltenyi Biotec) incubated with anti-CD4 conjugated to biotin (clone GK1.5, BioLegend) and anti-CD8α conjugated to biotin (clone 53-6.7, eBioscience), and CD11b^+^ myeloid cells were isolated from various tissue preparations using anti-mouse CD11b-coated magnetic MACS® MicroBeads (Miltenyi Biotec). All MACS beads-based isolations were performed according to the manufacturer’s instructions using MACS MS Cell Separation Columns (Miltenyi Biotec). Isolated cells were either lysed in sample buffer (50 mM Tris pH 6.8, 10% glycerol (v/v), 100 mM DTT, 2% SDS (w/v), bromophenol blue) immediately after isolation or cultured in RPMI-1640 + GlutaMAX supplemented with 10% (v/v) FCS and Penicillin/Streptomycin and treated with 10 μM MG132 in DMSO (Sigma Aldrich) as indicated, washed in PBS, and then lysed in sample buffer.

#### Quantitative Real-Time PCR

Equal numbers of BMDMs were seeded in 12-well plates 24 hr prior to experiments. Total RNA was extracted from BMDMs using RNeasy Mini Kit (QIAGEN, Hilden, Germany), and DNase digestion was performed on column with the RNase-Free DNase Set (QIAGEN, Hilden, Germany) according to the manufacturer’s protocol. Total RNA was reverse transcribed using Quantitect Reverse Transcription Kit (QIAGEN). RT-PCR was performed using QuantiFast SYBR Green RT-PCR Kit (QIAGEN) on a Viia7 Real-Time PCR Instrument (Applied Biosystems, Foster City, CA) with the primers indicated below. Each sample was run in duplicate. Results were normalized to those of 18S rRNA as internal housekeeping control using the 2^∧^(-ΔCt)-method. Primers were: 18S *rRNA* 5′-GTAACCCGTTGA-ACCCCATT-3′ and 5′-CCATCCAATCGGTAGTAGCG-3′; *Tnf* 5′-GGTCTGGGCCATAGAACTGA-3′ and 5′-CAGCCTCTTCTCATTCCTGC-3′; *Il6* 5′-TCTGAAGGACTCTGGCTTTG-3′ and 5′-GATGGATGCTACCAAACTGGA-3′; *Nfkbia* 5′-CCAAGTGCAGGAACGAGTCT-3′ and 5′-AAGGACGAGGAGTACGAGCA-3′; *Tnfaip3* 5′-TTCCTCAGGACCAGGTCAGT-3′ and 5′-AAGCTCGTGGCTCTGAAAAC-3′; *Hoip/Rnf31* 5′-TACGGTTGTATGGCTATA-3′ and 5′-GTATTCATCTGGTTCCTC-3′; *Hoil-1/Rbck1* 5′-GCACTTTCATCAACAAAC-3′ and 5′-AGGTATCTGGTAGGTCTC-3′; *Sharpin* 5′- GAACTGGTATTGTCTTGTGTA-3′ and 5′-AGAAGGCAAGGATGAACT-3′.

#### Immunoblotting

For BMDMs, equal numbers of cells were lysed directly in sample buffer (50 mM Tris pH 6.8, 10% glycerol (v/v), 100 mM DTT, 2% SDS (w/v), bromophenol blue), sonicated for 5 s (microtip) and boiled for 2 min. Mouse tissues were lysed in RIPA buffer (50 mM Tris pH 7.4, 1% NP-40 (v/v), 0.5% deoxycholate (w/v), 0.1% SDS (w/v), 150 mM NaCl, 2 mM EDTA, 5 mM MgCl_2_) for 15 min at 20 Hz on a TisssueLyser II (QIAGEN). Samples were then treated with Benzonase (Novagen, Madison, WI) for 30 min at 4°C for and then sonicated for 5 min in 10 s pulses. Samples were cleared by centrifugation and protein concentration was measured using BCA assay (Thermo Scientific). Proteins were resolved on 4%–12% Bis-Tris gels (Invitrogen, Carlsbad, CA) and transferred to nitrocellulose membranes using the iBlot system (Invitrogen), a HEP-1 Owl Panther semidry electroblotter (Thermo Scientific), or the Trans-Blot Turbo transfer system (Bio-Rad, Hercules, CA). Membranes were blocked in 5% milk in PBS-T (PBS + 0.1% (v/v) Tween-20) for 30 min and incubated with primary antibodies in PBS-T + 3% BSA at 4°C overnight, washed in PBS-T, incubated at room temperature for 1 hr with anti-rabbit IgG-HRP or anti-mouse IgG-HRP, washed, and visualized using Amersham Western Blotting Detection Reagent (GE Healthcare) or SuperSignal West Femto Maximum Sensitivity Substrate (Thermo Scientiric). Primary antibodies recognized OTULIN (14127, Cell Signaling Technology), IκBα (9242, Cell Signaling Technology), phospho-p65/RelA (pS563) (3033, Cell Signaling Technology), p65/RelA (8242, Cell Signaling Technology), A20 (4625, Cell Signaling Technology), NEMO/IKKγ (8330, Santa Cruz Biotechnology), GAPDH (AM4300, Ambion), Met1-linked/linear ubiquitin (LUB9, AB130, LifeSensors), Met1-linked/linear ubiquitin (1E3, MABS199, Millipore), Lys63-linked ubiquitin (Apu3, 05-1308, Millipore), Lys48-linked ubiquitin (Apu2, 05-1307, Millipore), Ubiquitin (NB300-130, Novus Biologicals), HOIP/RNF31 (46322, Abcam), mouse HOIP (kind gift from Professor Kazuhiro Iwai, Kyoto University (Tokunaga et al., 2011)), HOIL-1/RBCK1 (NBP2-27105, Novus Biologicals), HOIL-1/RBCK1 (sc-49718, Santa Cruz Biotechnology), SHARPIN (14626-1-AP, Proteintech), NIK (sc-8417, Santa Cryz Biotechnology), phospho-IRF-3 (pS396) (4947, Cell Signaling Technology), IRF-3 (sc-9082, Santa Cruz Biotechnology), CYLD (sc-74435, Santa Cruz Biotechnology), p38 (M138, ab31828, Abcam), phospho-p38 (pT180/pY182) (ERP18120, ab195049, Abcam), and Actin (C4, MAB1501R, Millipore). Secondary HRP-coupled antibodies were from GE Healthcare (NA931 and NA934). Densitometry analyses of immunoblots were performed using the Fiji software (Schindelin et al., 2012). To analyze IκBα stability, the ratio between the absolute IκBα signal intensity for each sample and the absolute Actin signal intensity for each samples was calculated. This ratio was then normalized to the “0” sample within each group. Immunoblot data are representative of at least two independent experiments.

#### MTT Reduction Assay for Cell Viability

MTT reduction assay was performed as previously described (Damgaard et al., 2013). Briefly, equal numbers of LysMCre-*Otulin*^+/flox^ and LysMCre-*Otulin*^*Lac*Z/flox^ BMDMs were seeded (as two biological replicas) in 96-well plates 24 hr before treatment. Cells were treated as indicated with recombinant human TNF (100 ng/mL; R&D Systems), ultrapure LPS from *E. coli* K12 (100 ng/mL; Invivogen, San Diego, CA), or Staurosporine (1 μM; Sigma-Aldrich) for 10 hr. Medium was aspirated and 100 μL fresh medium was added together with 25 μL 3-(4,5-dimethylthiazol-2-yl)-2,5-diphenyltetrazoliym bromide (MTT; 5 mg/mL dissolved in PBS (Sigma)) and cells were left to incubate for 2 hr at 37°C. Afterward, 100 μL solubilization buffer (20% SDS (w/v) dissolved in 50% *N,N*-dimethylformamide) was added and samples were left to incubate overnight. Absorbance at 590 nm was read with a reference filter of 620 nm. Individual experiments were performed in duplicate. Data were normalized to untreated LysMCre-*Otulin*^+/flox^ samples.

#### Kaplan-Meier Curve

The Kaplan-Meier curve for the LysMCre-*Otulin* mice were produced using Graphpad Prism 6.

#### Cell Culture and Pull-Downs

HEK293 cells were transfected with pEGFP-N1-OTULIN-WT or pEGFP-N1-OTULIN-L272P, encoding human OTULIN and its variant, using FuGENE HD (Promega, Madison, WI). Medium was changed 16 hr after transfection, and cells were then kept at 32°C for another 24 hr. Cell were lysed in 25 mM Tris-HCl pH 7.4, 150 mM NaCl, 1 mM DTT, 0.5% NP40 and protease inhibitor cocktail (Roche) on ice for 20 min. Lysate was cleared by centrifugation at 16,000 x *g* for 20 min, and lysate was incubated with agarose-coupled GFP-Trap A beads (ChromoTek GmbH, Martinsried, Germany) for 90 min at 4°C on rotation to precipitate GFP-tagged OTULIN. Bound GFP-OTULIN was eluted by boiling beads in 2x sample buffer, and the precipitated proteins were resolved and analyzed by SDS-PAGE and immunoblotting.

#### Patient Cell Samples

Buffy coat cells from blood samples from patient V:2 and anonymized healthy, age-matched controls were depleted of dead cells using the Dead Cell Removal kit (Miltenyi Biotec) according to the manufacturer’s instructions. Live cells were washed in PBS, pelleted by centrifugation, and lysed in sample buffer (50 mM Tris pH 6.8, 10% glycerol (v/v), 100 mM DTT, 2% SDS (w/v), bromophenol blue) before they were subjected to analysis by immunoblot. This experiment was repeated with two independent samples from patient V:2.

#### Expression and Purification of OTULIN

Human OTULIN constructs were expressed in *E. coli* strain Rosetta2 (DE3) pLacI. Cells were grown at 37°C in 2 xTY medium containing 30 mg/mL kanamycin and 34 mg/mL chloramphenicol to an OD_600_ of 0.8. The cultures were cooled down to 18°C before induction with 400 μM IPTG and harvested 20 hr post induction. Cells were resuspended and lysed by sonication in lysis buffer (20 mM Tris pH 7.4, 300 nM NaCl, 2 mM β-mercaptoethanol, 40 mM imidazole, DNase I, lysozyme, protease inhibitor cocktail (Roche)). OTULIN was purified by immobilized metal affinity chromatography using a HisTrap HP column (GE Healthcare Life Sciences). The 6-His tag was cleaved by overnight incubation with 3C protease, in a dialysis buffer (20 mM Tris pH 8.0, 4 mM DTT). The protein was further purified by anion exchange chromatography (ResourceQ, GE Healthcare Life Sciences) and the eluted OTULIN was subjected to size exclusion chromatography (HiLoad 16/60 Superdex 75, GE Healthcare Life Sciences) in buffer containing 20 mM Tris pH 8.0, 175 mM NaCl, 4 mM DTT.

#### Qualitative DUB Linkage Specificity Assay

Qualitative deubiquitination assays were performed as previously described (Keusekotten et al., 2013). Briefly, OTULIN^WT^ and OTULIN^L272P^ at several concentrations were diluted in 25 mM Tris pH 7.4, 150 mM NaCl, and 10 mM DTT and incubated with 1 μM di- or tetraUb in DUB buffer (50 mM Tris pH 7.4, 50 mM NaCl, 5 mM DTT) at 37°C. Samples were taken at different time points and mixed with 4 x SDS sample buffer to stop the reaction. They were resolved by SDS-PAGE and visualized by silver staining (BioRad SilverStain Plus kit). Qualitative DUB assay on diUb were repeated three times, and the assay were performed once on tetraUb.

#### Binding Studies

To measure binding affinities of OTULIN^C129A^ and OTULIN^C129A/L272P^ to Met1-diUb, fluorescence anisotropy experiments were performed as previously described (Keusekotten et al., 2013). In brief, 10 μL of 100 nM FlAsH-tagged Met1-diUb were dispensed in a 384-well Corning plate. Serial dilutions of OTULIN^C129A^ and OTULIN^C129/L272P^ were prepared in FlAsH buffer (20 mM Tris pH 7.4, 150 mM NaCl, 2 mM β-mercaptoethanol, 0.1 mg/mL bovine serum albumin) and 10 μL were added to FlAsH-tagged Met1-diUb containing wells. Fluorescence polarization was recorded on a PheraStar plate reader (BMG Labtech) using an optics module with λ_*ex*_ = 485 nm and λ_*em*_ = 520 nm, and values were fitted to a one-site total binding model using Graphpad Prism 6 to derive binding constants (K_D_).

#### Circular Dichroism Experiments

Circular dichroism (CD) measurements were performed on a Jasco-J815 spectropolarimeter (Jasco International Co. Ltd., Tokyo, Japan), using a spectral band width of 1 nm and a cell path-length of 0.1 cm. OTULIN^WT^ and OTULIN^L272P^ were re-buffered in PBS at 0.38 mg/mL prior to the experiment. The far UV CD spectra were measured from 260-195 nm and were corrected for buffer contribution by baseline subtraction.

#### Nano-DSF Thermal Unfolding Experiments

Nano-DSF (differential scanning fluorimetry) measurements were performed using a Prometheus NT.48 instrument (NanoTemper Technologies GmbH, Germany). Experiments on OTULIN^WT^ and OTULIN^L272P^ were carried out at 0.11 mg/mL and 0.38 mg/mL (not shown). Samples were dialysed into PBS before measurement and 10 μL of each sample were loaded in UV capillaries (NanoTemper Technologies). Temperature gradient was set at 2.5°C/min in a range from 20 to 90°C. Protein unfolding was measured by detection of change in tryptophan fluorescence at emission wavelengths of 330 and 350 nm, dependent on temperature gradient. T_m_’s were calculated according to the manufacturer’s instructions. Results were confirmed by differential scanning calorimetry and CD thermal melt experiments (data not shown).

### Quantification and Statistical Analysis

Data are presented as mean ± SEM unless otherwise indicated in figure legends. Sample number (n) indicates the number of independent biological samples in each experiment. Sample numbers and experimental repeats are indicated in figures and figure legends or methods section above. Data were analyzed using the two-sided nonparametric Mann-Whitney U test of the null hypothesis of continuous data unless otherwise indicated in figure legends or method details. Data analysis was not blinded. Differences in means were considered statistically significant at p < 0.05. Significance levels are: ^∗^ p < 0.05; ^∗∗^ p < 0.01; ^∗∗∗^ p < 0.001; ^∗∗∗∗^ p < 0.0001; n.s., non-significant. Analyses were performed using the Graphpad Prism 6.0 software.

### Data and Software Availability

#### Data Resources

Due to restrictions from the patient consent approved by the research ethics committee it is not be possible to deposit complete exome sequencing data in a public repository, but the data could be made available to interested researchers by contacting the authors.

## Author Contributions

D.K., A.N.J.M., and E.R.M. conceived the project. R.B.D., J.W., A.N.J.M and D.K. designed in vivo experiments, and R.B.D. performed and analyzed the experiments assisted by J.W. R.B.D. designed and performed the in vitro experiments and analyzed the data. E.R.M., D.M., N.V.M., and H.L.T. were responsible for human clinical and molecular genetic studies. P.M.-C. performed and analyzed the biochemical and biophysical experiments, to which also P.R.E. contributed. R.B.D. and D.K. wrote the paper with input from all authors.

## Supplemental Information

Document S1. Tables S1 and S3–S8

Table S2. Rare Homozygous Variants in ORAS Patients, Related to Figure 1 Details of rare homozygous variants (allele frequency < 0.01) identified by exome sequencing in an affected individual. The three variants within the linked autozygous region on chromosome 5p15 are shaded.
