# Supplementary material for: The Deubiquitinase OTULIN Is an Essential Negative Regulator of Inflammation and Autoimmunity
Source: Cell. 2016 Aug 25;166(5):1215–1230.e20. doi: 10.1016/j.cell.2016.07.019 (PMC5002269; doi:10.1016/j.cell.2016.07.019)
Supplement: Document S1. Tables S1 and S3–S8 [file mmc1.pdf]

Cell, Volume *166*

Supplemental Information

The Deubiquitinase OTULIN Is an Essential Negative Regulator of Inflammation and Autoimmunity

Rune Busk Damgaard, Jennifer A. Walker, Paola Marco-Casanova, Neil V. Morgan, Hannah L. Titheradge, Paul R. Elliott, Duncan McHale, Eamonn R. Maher, Andrew N.J. McKenzie, and David Komander

Table S1 (related to **Figure 1**). **Serum immunoglobulin (Ig) levels from ORAS patients.**

Tabulated serum immunoglobulin (Ig) levels from ORAS patients IV:3, IV:4, and V:2. Age of the patient at the time of sampling is indicated. Concentration of Ig is mg/mL with age-corrected normal range shown in brackets. Red shading indicates measurements that exceed the normal range.

Patient	Time of sample	Ig	Concentration [mg/mL] (normal range)	
**IV:3**				
	Age 2 months	IgG	8.48 (2.1-7.7)	
		IgA	1.01 (0.05-0.4)	
		IgM	1.07 (0.15-0.7)	
	Age 3 months	IgG	6.18 (2.4-8.8)	
			IgG1	6.18 (2.2-4.95)
			IgG2	0.57 (0.4-1.65)
			IgG3	0.99 (0.04-0.23)
			IgG4	<0.11 (0.01-1.3)
		IgA	0.83 (0.1-0.6)	
		IgM	1.38 (0.2-1.0)	
**IV:4**				
	Age 4 days	IgG	5.58 (5.0-17)	
		IgA	<0.07 (0.01)	
		IgM	0.11 (0.05-0.2)	
	Age 2 years and 10 months	IgG	13.5 (3.1-13.8)	
		IgA	1.95 (0.3-1.2)	
		IgM	1.05 (0.5-2.2)	
**V:2**				
	Pre-infliximab	IgG	11.0 (3.6-15.2)	
		IgA	2.61 (0.35-1.39)	
		IgM	3.35 (0.43-1.9)	
	Post-infliximab (4 years)	IgG	10.18 (4.9-16.1)	
		IgA	0.93 (0.4-2.0)	
		IgM	1.46 (0.5-2.0)	

Table S3 (related to **Figure 1**). **Pathogenic genetic variants in ORAS patients.**

Details of 50 genetic variants detected in the exome from an individual affected by ORAS and annotated as pathogenic in the ClinVar database (http://www.ncbi.nlm.nih.gov/clinvar/). In the majority of cases these variants had a maximum allele frequency (AF_MAX) >0.1 in normal control databases of different populations and none were considered as a likely cause of the disease phenotype.

Position chr:nucleotide	Change	Genotype	gene	effect	dbsnp	clinVar significance	AF_MAX
1:100672060	T>C	HOMO	DBT	missense variant	rs12021720	pathogenic	0.966783
1:161599693	T>C	HET	FCGR3B	missense variant	rs76714703	pathogenic	0.5605
1:98348885	G>A	HOMO	DPYD	missense variant	rs1801265	pathogenic	0.942308
10:54531242	G>A	HET	MBL2	missense variant	rs5030737	pathogenic	0.065963
10:70645376	A>C	HET	STOX1	missense variant	rs10509305	pathogenic	0.237569
11:113270828	G>A	HET	ANKK1	missense variant	rs1800497	pathogenic	0.388112
11:18290859	C>T	HET	SAA1	missense variant	rs1136743	pathogenic	0.608179
11:68846399	A>T	HET	TPCN2	missense variant	rs35264875	pathogenic	0.21504
11:68846399	A>T	HET	TPCN2	missense variant	rs35264875	pathogenic	0.21504
11:68855363	G>A	HET	TPCN2	missense variant	rs3829241	pathogenic	0.362797
12:121176083	G>A	HET	ACADS	missense variant & splice region variant	rs1799958	pathogenic	0.314917
12:121437382	A>G	HOMO	HNF1A	missense variant	rs1169305	pathogenic	1
12:122295335	T>C	HOMO	HPD	missense variant	rs1154510	pathogenic	0.945122
14:21790040	G>T	HET	RPGRIP1	missense variant	rs10151259	pathogenic	0.240106
15:28230318	C>T	HET	OCA2	missense variant	rs1800407	pathogenic	0.079156
16:48258198	C>T	HOMO	ABCC11	missense variant	rs17822931	pathogenic	0.90035
16:56548501	C>T	HOMO	BBS2	missense variant	rs4784677	pathogenic	1
17:12899902	C>T	HET	ELAC2	missense variant	rs5030739	pathogenic	0.046174
17:12915009	G>A	HOMO	ELAC2	missense variant	rs4792311	pathogenic	0.327177
17:42453065	A>C	HET	ITGA2B	missense variant	rs5911	pathogenic	0.432927
17:48437456	C>G	HOMO	XYLT2	missense variant	rs6504649	pathogenic	0.41029
2:219755011	T>A	HOMO	WNT10A	missense variant	rs121908120	pathogenic	0.019789
20:43280227	C>T	HET	ADA	missense variant & NMD transcript variant	rs11555565	pathogenic	0.067282
22:42522613	G>C	HOMO	CYP2D6	missense variant	rs1135840	pathogenic	0.519337
22:42523943	A>G	HOMO	CYP2D6	splice region variant & intron variant & NMD transcript variant	rs16947	pathogenic	0.847902
22:42524243	CT>C	HET	CYP2D6	Frameshift variant & feature truncation	rs35742686	pathogenic	0.021108
22:46731689	G>T	HET	TRMU	missense variant & NMD transcript variant	rs11090865	pathogenic	0.22561
3:122003757	G>T	HET	CASR	missense variant	rs1801725	pathogenic	0.138522
4:102751076	G>A	HET	BANK1	missense variant	rs10516487	pathogenic	0.28628
4:187113041	C>G	HET	CYP4V2	missense variant	rs1055138	pathogenic	0.536939
5:118811533	G>A	HET	HSD17B4	missense variant	rs25640	pathogenic	0.497238
5:149212243	G>C	HET	PPARGC1B	missense variant	rs7732671	pathogenic	0.160569
5:35861068	T>C	HET	IL7R	missense variant & NMD transcript variant	rs1494558	pathogenic	0.745935
5:35861068	T>C	HET	IL7R	missense variant	rs1494558	pathogenic	0.745935
5:35871190	G>A	HET	IL7R	missense variant & NMD transcript variant	rs1494555	pathogenic	0.902439
5:73981270	T>C	HOMO	HEXB	missense variant	rs820878	pathogenic	1

6:29080344	G>A	HET	OR2J3	missense variant	rs3749977	pathogenic	0.619919
7:138417791	A>G	HET	ATP6V0A4	missense variant	rs3807153	pathogenic	0.219512
7:142458451	A>T	HET	PRSS1	missense variant	rs111033566	pathogenic	N/A
8:21976710	T>C	HET	HR	missense variant	rs7014851	pathogenic	0.284553
9:136301982	C>G	HET	ADAMTS13	missense variant	rs2301612	pathogenic	0.437995
MT:6480	G>A	HOMO	MT-CO1	missense variant	rs199476128	pathogenic	N/A
11:126162843	C>T	HET	TIRAP	missense variant & NMD transcript variant	rs8177374	pathogenic & protective	0.167546
1:196659237	C>T	HET	CFH	missense variant	rs1061170	pathogenic & risk factor	0.931818
1:223285200	G>A	HOMO	TLR5	stop gained	rs5744168	pathogenic & risk factor	0.055409
12:121416650	A>C	HET	HNF1A	Splice region variant & intron variant & NMD transcript variant	rs1169288	pathogenic & risk factor	0.424825
16:27374180	T>C	HET	IL4R	missense variant	rs1805015	pathogenic & risk factor	0.461382
16:69745145	G>A	HET	NQO1	missense variant	rs1800566	pathogenic & risk factor	0.424825
22:18901004	C>T	HOMO	PRODH	missense variant	rs450046	pathogenic & risk factor	0.963287
7:150696111	T>G	HET	NOS3	missense variant	rs1799983	pathogenic & risk factor	0.936992

Table S4 (related to **Figure 2**). **Serum cytokine and chemokine concentrations in CreERT2-*Otulin*$^{+/flox}$ and CreERT2-*Otulin*$^{LacZ/flox}$ bone marrow chimeric mice.**

Tabulated serum cytokine and chemokine concentrations from terminal bleeds of CreERT2-*Otulin*$^{+/flox}$ and CreERT2-*Otulin*$^{LacZ/flox}$ chimeras and vehicle treated controls on day 5 measured by Luminex multiplex analysis. Significance level indicates the *P*-value of the difference in means between tamoxifen-treated +/flox and *Lac*Z/flox chimeras. Green indicates upregulated cytokines/chemokines. Orange indicates downregulated cytokines/chemokines. n.s., non-significant. Data were pooled from two independent experiments. Data are presented as mean ± s.e.m., and n represents number of mice.

	Concentration (mean ± s.e.m.)				
	Tamoxifen		Vehicle		
**Analyte**	**+/flox, n=13**	***LacZ*/flox, n=14**	**+/flox, n=2**	***LacZ*/flox, n=2**	**Significance level**
G-CSF	**470.30** ± 139.60	**55216.0** ± 8756.0	**227.50** ± 72.89	**168.10** ± 62.95	****
GM-CSF	**669.60** ± 49.08	**909.00** ± 82.56	**842.5** ± 68.65	**793.80** ± 171.20	n.s.
IFN-γ	**4.92** ± 0.49	**7.56** ± 0.57	**3.92** ± 0.72	**3.92** ± 0.72	n.s.
IL-1α	**477.60** ± 155.20	**248.10** ± 41.80	**182.80** ± 106.2	**181.4** ± 119.50	n.s.
IL-1β	**34.86** ± 5.30	**38.91** ± 2.93	**17.26** ± 5.32	**36.27** ± 11.12	n.s.
IL-2	**3.69** ± 0.30	**3.70** ± 0.34	**2.49** ± 0.30	**3.18** ± 0.78	n.s.
IL-4	**2.10** ± 0.50	**1.67** ± 0.25	**2.13** ± 0.99	**1.78** ± 0.77	n.s.
IL-5	**41.01** ± 5.35	**13.75** ± 1.637	**50.36** ± 8.01	**37.20** ± 3.34	****
IL-6	**7.95** ± 2.87	**336.60** ± 61.09	**23.89** ± 18.72	**5.66** ± 2.94	****
IL-7	**7.54** ± 1.10	**10.79** ± 0.98	**6.13** ± 0.77	**9.27** ± 0.80	n.s.
IL-9	**99.05** ± 52.0	**66.48** ± 28.89	**87.63** ± 87.63	**84.08** ± 84.08	n.s.
IL-10	**7.60** ± 0.84	**11.79** ± 1.10	**6.03** ± 0.48	**4.58** ± 0.0	n.s.
IL-12p40	**7.85** ± 0.97	**12.66** ± 2.53	**3.69** ± 0.13	**6.63** ± 0.77	n.s.
IL-12p70	**37.80** ± 9.51	**50.28** ± 7.10	**32.93** ± 14.49	**47.06** ± 11.24	n.s.
IL-13	**167.7** ± 14.45	**257.10** ± 22.29	**203.20** ± 40.99	**172.6** ± 40.63	n.s.
IL-15	**20.81** ± 3.67	**38.78** ± 5.75	**13.00** ± 13.00	**15.80** ± 10.21	n.s.
IL-17	**6.81** ± 1.20	**7.91** ± 1.07	**14.47** ± 8.18	**10.08** ± 4.51	n.s.
IP-10	**669.60** ± 49.08	**909.00** ± 82.56	**842.50** ± 68.65	**793.80** ± 171.20	n.s.
KC	**85.53** ± 20.18	**450.50** ± 90.12	**11.27** ± 0.09	**13.25** ± 3.47	****
MCP-1	**167.50** ± 16.12	**353.20** ± 35.05	**111.90** ± 11.45	**154.00** ± 22.24	****
MIP-1α	**111.70** ± 12.64	**143.40** ± 6.94	**98.10** ± 11.52	**99.82** ± 18.26	n.s.
MIP-1β	**49.63** ± 11.95	**57.89** ± 5.47	**68.29** ± 38.41	**53.70** ± 23.82	n.s.
MIP-2	**110.50** ± 4.35	**109.60** ± 6.81	**96.08** ± 18.75	**110.90** ± 21.08	n.s.
RANTES	**13.04** ± 1.52	**14.74** ± 0.92	**11.27** ± 0.09	**13.25** ± 3.47	n.s.
TNF	**13.70** ± 2.14	**21.58** ± 1.67	**9.67** ± 0.77	**11.88** ± 1.44	**

Table S5 (related to **Figure 3**). **Serum cytokine and chemokine concentrations in CD4Cre-*Otulin*$^{+/flox}$ and CD4Cre-*Otulin*$^{del/flox}$ mice**

Tabulated serum cytokine and chemokine concentrations from terminal bleeds of CD4Cre-*Otulin*$^{+/flox}$ and CD4Cre-*Otulin*$^{del/flox}$ mice measured by MAGPIX multiplex analysis. Significance level indicates the *P*-value of the difference in means between +/flox and del/flox mice. Green indicates upregulated cytokines/chemokines. Orange indicates downregulated cytokines/chemokines. n.s., non-significant. Data are presented as mean ± s.e.m., and n represents number of mice.

	Concentration, pg/mL (mean ± s.e.m.)		
**Analyte**	**+/flox, n=6**	**del/flox, n=6**	**Significance level**
G-CSF	472 ± 133.70	382 ± 67.35	n.s.
GM-CSF	67.03 ± 5.22	65.06 ± 5.15	n.s.
IFN-γ	4.89 ± 0.63	8.59 ± 1.54	n.s.
IL-1α	225.20 ± 48.31	173.40 ± 18.44	n.s.
IL-1β	20.70 ± 1.16	21.05 ±2.71	n.s.
IL-2	1.63 ± 0.25	1.87 ± 0.31	n.s.
IL-4	0.69 ± 0.16	1.12 ± 0.16	n.s.
IL-5	13.75 ± 2.80	20.30 ± 3.53	n.s.
IL-6	8.31 ± 5.01	21.75 ± 6.05	n.s.
IL-7	5.12 ± 0.39	8.49 ± 2.34	n.s.
IL-9	100.30 ± 15.56	111.10 ± 23.37	n.s.
IL-10	11.80 ± 1.49	10.60 ±1.26	n.s.
IL-12p40	10.55 ± 2.26	12.05 ± 2.58	n.s.
IL-12p70	16.86 ± 3.59	18.77 ± 2.37	n.s.
IL-13	114.10 ± 10.03	108.80 ± 10.51	n.s.
IL-15	66.69 ± 5.59	88.19 ± 16.51	n.s.
IL-17	4.41 ± 0.38	5.06 ± 1.08	n.s.
IP-10	213.90 ± 20.94	278.30 ± 27.83	n.s.
KC	123.10 ± 16.81	142.30 ± 12.45	n.s.
MCP-1	70.11 ± 3.41	69.87 ± 6.76	n.s.
MIP-1α	72.95 ± 6.63	66.20 ± 5.47	n.s.
MIP-1β	71.37 ± 5.39	85.82 ± 9.37	n.s.
MIP-2	108.60 ± 8.87	97.24 ± 6.23	n.s.
RANTES	33.36 ± 3.67	36.37 ± 4.79	n.s.
TNF	5.90 ± 0.66	6.58 ± 0.84	n.s.

Table S6 (related to **Figure 3**). **Serum cytokine and chemokine concentrations in MB1Cre-*Otulin*$^{+/flox}$ and MB1Cre-*Otulin*$^{LacZ/flox}$ mice.** Tabulated serum cytokine and chemokine concentrations from terminal bleeds of MB1Cre-*Otulin*$^{+/flox}$ and MB1Cre-*Otulin*$^{LacZ/flox}$ mice measured by MAGPIX multiplex analysis. Significance level indicates the *P*-value of the difference in means between +/flox and del/flox mice. Green indicates upregulated cytokines/chemokines. Orange indicates downregulated cytokines/chemokines. n.s., non-significant. Data are presented as mean ± s.e.m., and n represents number of mice.

	Concentration, pg/mL (mean ± s.e.m.)		
**Analyte**	**+/flox, n=7**	***LacZ*/flox, n=7**	**Significance level**
G-CSF	382.10 ± 104.30	195.30 ± 52.50	n.s.
GM-CSF	70.17 ± 6.52	44.31 ± 10.87	n.s.
IFN-γ	5.96 ± 0.80	4.50 ± 0.87	n.s.
IL-1α	344.40 ± 71.07	385.90 ± 94.17	n.s.
IL-1β	26.59 ± 6.29	17.40 ± 2.09	n.s.
IL-2	2.08 ± 0.31	1.12 ± 0.18	*
IL-4	0.68 ± 0.10	0.42 ± 0.12	n.s.
IL-5	20.61 ± 6.56	13.55 ± 2.50	n.s.
IL-6	16.69 ± 13.23	4.13 ± 1.52	n.s.
IL-7	23.12 ± 12.12	10.69 ± 3.81	n.s.
IL-9	81.60 ± 17.04	93.25 ± 21.98	n.s.
IL-10	30.24 ± 19.94	6.91 ± 1.31	n.s.
IL-12p40	6.73 ± 2.52	6.50 ± 2.55	n.s.
IL-12p70	14.51 ± 2.41	8.48 ± 3.77	n.s.
IL-13	115.10 ± 17.32	112 ± 18.97	n.s.
IL-15	87.39 ± 10.94	71.03 ± 9.04	n.s.
IL-17	19.29 ± 16.61	2.28 ± 0.49	n.s.
IP-10	207.70 ± 14.76	224.80 ± 37.63	n.s.
KC	124.90 ± 22.66	101.90 ± 15.74	n.s.
MCP-1	71.70 ± 7.63	58.56 ± 6.29	n.s.
MIP-1α	81.64 ± 19.47	62.16 ± 10.30	n.s.
MIP-1β	55.18 ± 18.55	38.87 ± 11.42	n.s.
MIP-2	97.23 ± 3.58	83.28 ± 9.91	n.s.
RANTES	29.32 ± 5.74	16.34 ± 3.21	n.s.
TNF	5.49 ± 0.66	4.40 ± 0.83	n.s.

Table S7 (related to **Figure 3**). **Serum cytokine and chemokine concentrations in LysMCre-*Otulin*$^{+/flox}$ and LysMCre-*Otulin*$^{LacZ/flox}$ mice.** Tabulated serum cytokine and chemokine concentrations from terminal bleeds of sex- and age-matched 4 - 9 month old LysMCre-*Otulin*$^{+/flox}$ and LysMCre-*Otulin*$^{LacZ/flox}$ mice measured by MAGPIX multiplex analysis. Significance level indicates the *P*-value of the difference in means between LysMCre-*Otulin*$^{+/flox}$ and LysMCre-*Otulin*$^{LacZ/flox}$ mice. Green indicates upregulated cytokines/chemokines. n.s., non-significant. Data were pooled from three independent experiments. Data are presented as mean ± s.e.m., and n represents number of mice.

	Concentration, pg/mL (mean ± s.e.m.)		
**Analyte**	**+/flox, n=12**	***LacZ*/flox, n=12**	**Significance level**
G-CSF	**195.50** ± 49.79	**3285.00** ± 968.50	****
GM-CSF	**17.96** ± 7.66	**108.20** ± 28.01	***
IFN-γ	**0.97**± 0.29	**16.09** ± 6.75	**
IL-1α	**851.20** ± 246.60	**465.60** ± 84.46	n.s.
IL-1β	**6.81** ± 1.51	**35.01** ± 4.08	****
IL-2	**0.53** ± 0.17	**7.06** ± 2.85	****
IL-4	**0.30** ± 0.13	**3.50** ± 1.04	**
IL-5	**8.97** ± 2.10	**20.43** ± 3.40	**
IL-6	**1.94** ± 1.34	**79.81** ± 26.76	***
IL-7	**7.71** ± 4.00	**7.29** ± 2.12	n.s.
IL-9	**30.39** ± 8.69	**149.8** ± 63.42	n.s.
IL-10	**2.87** ± 1.10	**20.03** ± 4.60	****
IL-12p40	**3.76** ± 1.24	**4.84** ± 2.38	n.s.
IL-12p70	**0.21** ± 0.17	**20.81** ± 7.28	***
IL-13	**86.84** ± 10.47	**229.6** ± 63.56	*
IL-15	**14.76** ± 10.34	**9.80** ± 3.96	n.s.
IL-17	**0.78** ± 0.41	**6.25** ± 2.67	n.s.
IP-10	**256.30** ± 16.23	**774.70** ± 49.75	****
KC	**89.69** ± 25.12	**164.8** ± 35.60	n.s.
MCP-1	**55.95** ± 4.97	**137.10** ± 14.20	****
MIP-1α	**16.15** ± 7.46	**174.50** ± 53.86	****
MIP-1β	**9.63** ± 5.20	**31.68** ± 13.49	n.s.
MIP-2	**92.14** ± 9.69	**70.53** ± 5.56	n.s.
RANTES	**7.87** ± 1.37	**14.24** ± 2.03	*
TNF	**4.79** ± 0.40	**20.58** ± 1.59	****

Table S8 (related to Key Resources Table). Primer sequences for quantitative RT-PCR.

Sequence-Based Reagents - Primers		
18S *rRNA* RT-qPCR F primer 5'-GTAACCCGTTGAACCCCATT-3'	This paper	N/A
18S *rRNA* RT-qPCR R primer 5'-CCATCCAATCGGTAGTAGCG-3'	This paper	N/A
Mouse *Tnf* RT-qPCR F primer 5'-GGTCTGGGCCATAGAACTGA-3'	This paper	N/A
Mouse *Tnf* RT-qPCR R primer 5'-CAGCCTCTTCTCATTCCTGC-3'	This paper	N/A
Mouse *Il6* RT-qPCR F primer 5'-TCTGAAGGACTCTGGCTTTG-3'	This paper	N/A
Mouse *Il6* RT-qPCR R primer 5'-GATGGATGCTACCAAACTGGA-3'	This paper	N/A
Mouse *Nfkbia* RT-qPCR F primer 5'-CCAAGTGCAGGAACGAGTCT-3'	This paper	N/A
Mouse *Nfkbia* RT-qPCR R primer 5'-AAGGACGAGGAGTACGAGCA-3'	This paper	N/A
Mouse *Tnfaip3* RT-qPCR F primer 5'-TTCCTCAGGACCAGGTCAGT-3'	This paper	N/A
Mouse *Tnfaip3* RT-qPCR R primer 5'-AAGCTCGTGGCTCTGAAAAC-3'	This paper	N/A
Mouse *Hoip/Rnf31* RT-qPCR F primer 5'-TACGGTTGTATGGCTATA-3'	This paper	N/A
Mouse *Hoip/Rnf31* RT-qPCR R primer 5'-GTATTCATCTGGTTCCTC-3'	This paper	N/A
Mouse *Hoil-1/Rbck1* RT-qPCR F primer 5'-GCACTTTCATCAACAAAC-3'	This paper	N/A
Mouse *Hoil-1/Rbck1* RT-qPCR R primer 5'-AGGTATCTGGTAGGTCTC-3'	This paper	N/A
Mouse *Sharpin* RT-qPCR F primer 5'-GAACTGGTATTGTCTTGTGTA-3'	This paper	N/A
Mouse *Sharpin* RT-qPCR R primer 5'-AGAAGGCAAGGATGAACT-3'	This paper	N/A
